# Straightforward and Controlled Synthesis of Porphyrin–Phthalocyanine–Porphyrin Heteroleptic Triple‐Decker Assemblies

**DOI:** 10.1002/chem.202002500

**Published:** 2020-07-27

**Authors:** Daniel González‐Lucas, Shazia C. Soobrattee, David L. Hughes, Graham J. Tizzard, Simon J. Coles, Andrew N. Cammidge

**Affiliations:** ^1^ School of Chemistry University of East Anglia Norwich Research Park Norwich NR4 7TJ United Kingdom; ^2^ UK National Crystallography Service Chemistry University of Southampton Southampton SO17 1BJ United Kingdom

**Keywords:** dyes/pigments, lanthanides, phthalocyanines, porphyrinoids, synthesis

## Abstract

A versatile and straightforward protocol is disclosed for controlled synthesis of complex lanthanide‐bridged heteroleptic porphyrin–phthalocyanine triple‐decker assemblies. Two porphyrins, linked by a flexible spacer chain of intermediate length, sequentially capture lanthanide ions and a phthalocyanine to efficiently form the triple‐decker complex. The bridge directs assembly, but also controls the mobility of the central macrocycle and further imparts a fully eclipsed arrangement of all three rings.

## Graphical abstract

**A versatile and straightforward** protocol is disclosed for the controlled synthesis of complex lanthanide‐bridged heteroleptic porphyrin–phthalocyanine triple‐decker assemblies (see scheme). The bridge directs assembly, but also controls the mobility of the central macrocycle and further imparts a fully eclipsed arrangement of all three rings.

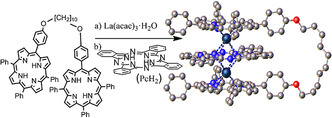

Natural and synthetic macrocyclic systems based on porphyrin **1** and phthalocyanine **2** parents (Figure 1) have been the focus of intense research over recent decades and continues to gain momentum.^1^ The assembly of these functional macrocycles into higher order covalent and supramolecular structures has been a fruitful goal and spectacular examples, often inspired by nature, have been reported.^2^

**Figure 1:**
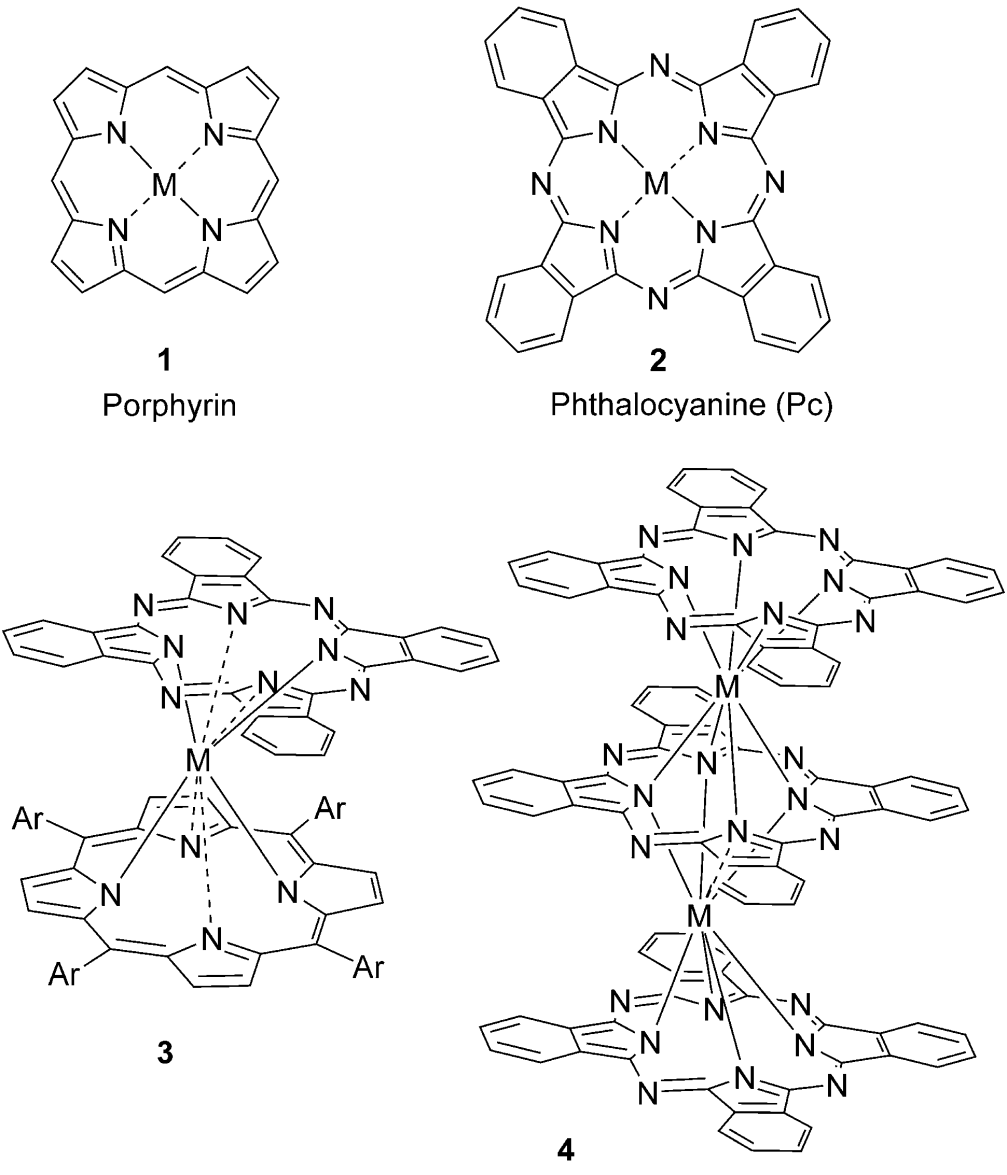
Parent structures porphyrin **1**, phthalocyanine **2**, a porphyrin–phthalocyanine double‐decker **3** and a phthalocyanine triple‐decker **4**.

The direct face‐to‐face assembly of porphyrins and phthalocyanines (i.e. without bridging ligands between the central metal ions) was first achieved for tin (IV) phthalocyanines,^3^ but most attention has focused on the fascinating lanthanide double‐ and triple‐decker sandwich complexes,^4^ not least because the assemblies introduce important new character such as Near IR absorption (due to π–π‐overlap between macrocycles) and molecular magnetism in some examples.^5^

A number of synthetic approaches to porphyrin–phthalocyanine multidecker systems have been investigated.^6^ They range from statistical strategies through sequential assembly and multistep synthesis. Although successful, with examples now including the syntheses of quadruple^7^ and quintuple^8^ systems, the strategies most often lead to mixtures and the products themselves are prone to subsequent re‐equilibration.

As part of a wider project to assemble high‐order, machine‐like constructs from functional porphyrin and phthalocyanine building blocks, we performed model studies on the construction of multidecker systems on a porphyrin dyad platform. The experiment and key discovery are shown in Scheme 1. Two porphyrin units were separated by a flexible chain of intermediate length (C_10_) and treated with lanthanum acac in hot octanol, monitoring the metalation by UV/Vis analysis of aliquots. When metalation was complete, 2 equivalents of phthalocyanine were added and reflux continued overnight.

**Scheme 1:**
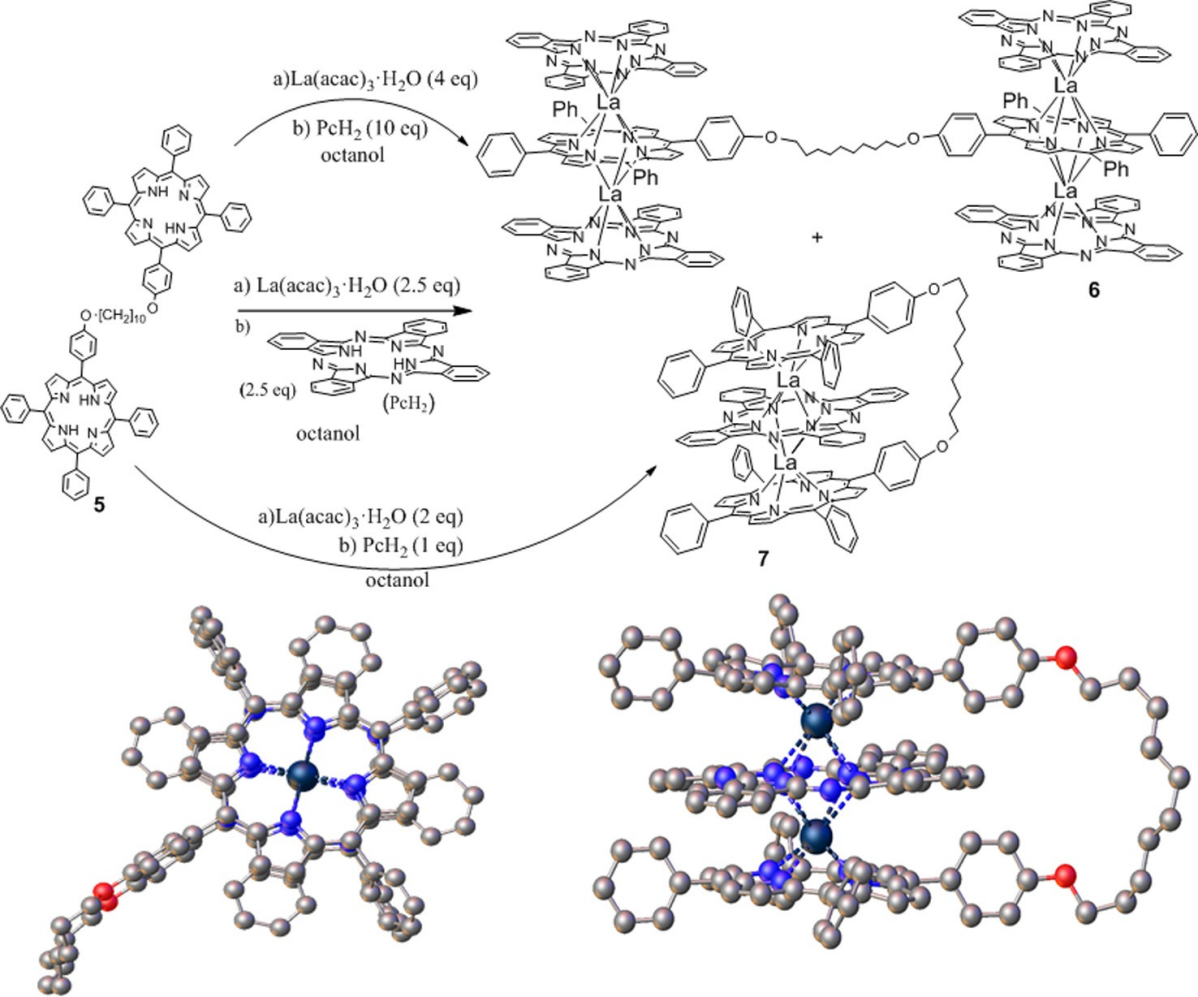
Controlled synthesis of lanthanum‐bridged porphyrin–phthalocyanine–porphyrin triple‐deckers (TD); inset, the X‐ray crystal structure of **7** showing direct alignment of the three macrocycles. Only one of the symmetry‐imposed disorder components is shown. Hydrogen atoms have been omitted for clarity.

The resulting reaction mixture contained mostly just two products (no starting materials) and separation and analysis revealed formation of bis‐triple decker **6** alongside the bridged triple‐decker **7**. There was no evidence for formation of the corresponding bis‐double‐decker. Repeating the reaction using excess phthalocyanine led to **6**, whereas using one equivalent of phthalocyanine led exclusively to **7** as a single product. The reaction is reproducible and the triple decker (TD) is robust. Full characterisation revealed several interesting features. X‐ray crystallography^9^ shows a symmetrical TD structure and minimal strain in the linking chain. However, the presence of the chain leads to an eclipsed arrangement of the macrocycles—the porphyrin rings are aligned, and this contrasts the staggered conformation observed in related complexes.5b, 10

In solution, absorption spectroscopy shows the TD arrangement with characteristic long wavelength absorptions extending to the near IR.6b, 6e NMR spectroscopy gives further insight, most importantly on the dynamics of the molecular gyroscope. In this parent system, at ambient temperature, ^1^H NMR spectroscopy reveals a single pair of signals for the central phthalocyanine macrocycle, implying rapid rotation on the NMR timescale.

The free rotation of the central phthalocyanine units was investigated alongside experiments to probe the versatility and tolerance of the synthetic method, using central Pc units with varying steric demand. Phthalocyanines **8** and **9** (Figure 2), bearing 8 *n*‐octyl‐substituents at the β‐11 and α‐11 sites, respectively were synthesised and employed in TD formation using the developed protocol. TD formation appeared smooth in both cases—each yielding a single major product that gave identical mass spectrometry results (cluster at *m*/*z=*3085 corresponding to the TD). However, further detailed characterisation revealed that the two complexes are significantly different. TD **10**, formed from the peripherally substituted phthalocyanine **8** gave an absorption spectrum that was essentially identical to parent TD **7**. Its ^1^H NMR spectrum also shows characteristic features of the parent system, but it is clear that the long sidechains are no longer able to pass the bridging chain and therefore they prevent free rotation of the central phthalocyanine macrocycle. In this situation the aromatic protons of the central macrocycle are no longer equivalent and are observed as 4 singlets (Figure 2, inset).

**Figure 2:**
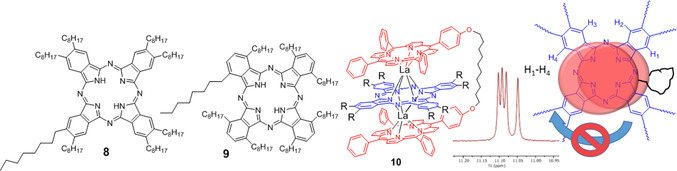
Triple‐decker formation from substituted phthalocyanines; peripherally substituted phthalocyanine **8** leads to stable TD **10** where rotation of the central macrocycle is prevented by the sidechains.

The assembly formed from non‐peripherally substituted phthalocyanine **9** is obtained as a pure, single compound but is significantly more complex. Both NMR and absorption spectroscopy show that a simple symmetrical assembly is not produced in this case. The NMR spectrum is complicated, but importantly the characteristic signals (around 10 ppm) previously observed in TDs for the inner phenyl protons of the porphyrins are absent. The absorption spectrum is also different and bears much closer similarity to the spectrum expected for a mixture of metalloporphyrin + phthalocyanine‐porphyrin double‐decker. The preliminary conclusion in this case is that the added steric demand of the central phthalocyanine, in which the sidechains are displaced above/below the macrocycle plane,^11^, ^12^ prevents symmetrical TD formation. The complex is unsymmetrical, with the central phthalocyanine likely to equilibrate in the sandwich between the two outer metalloporphyrins.

A rigid central phthalocyanine with intermediate steric bulk, **11** (Figure 3), bearing dimethyl dioxirane units at β‐sites, was selected for more detailed investigation. TD formation using porphyrin dyad **5** was again straightforward and the room‐temperature ^1^H NMR spectrum shows a symmetrical TD is formed. Separate Ar‐H signals, and two distinct singlets for the dioxirane units, clearly indicate restricted rotation. Spectra were recorded in toluene between 298–375 K. As the temperature is increased, the signals for the porphyrin *meso*‐phenyl substituents become broadened due to rotation, but the central Pc retains resolved signals for both Ar−H and methyl groups. The TD complex itself therefore is robust and the central phthalocyanine ring, in this case, is unable to undergo free rotation by passing the bridge.

**Figure 3:**
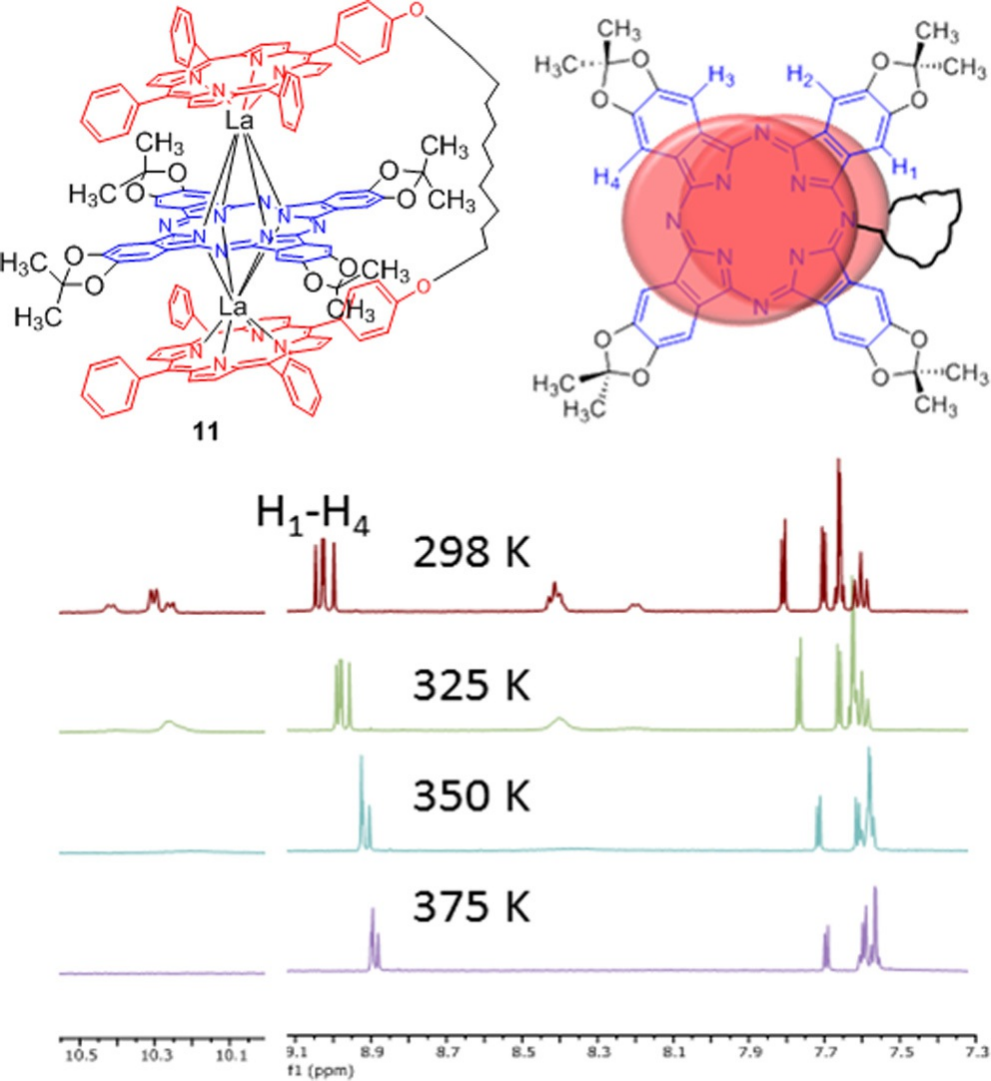
Variable temperature ^1^H NMR spectra for TD **11**.

A particular attraction of the lanthanide‐bridged double and triple decker assemblies is the potential for generation of (single molecule) magnetic materials through selection of alternative ions from the lanthanide series. Ion size contracts across the lanthanide series so the combination of simple synthesis protocol alongside the strain and conformational constraints imposed by the link chain has the potential to offer control in the construction of bespoke TD systems. Initial experiments have been performed and indicate that the systems do indeed offer excellent potential for selectivity (Scheme 2). Representative lanthanide (III) acac salts with reducing ionic radii were selected and used in TD formation using a modified version of the conditions established for the lanthanum‐bridged systems described above. Assembly and isolation of the praseodymium (**12**), neodymium (**13**), samarium (**14**) and europium (**15**) TDs proceeded smoothly in a single unoptimised operational step by directly heating a mixture of dyad **5**, lanthanide salt and PcH_2_ in octanol (radii Pr^3+^=99 pm, Nd^3+^=98 pm, Sm^3+^=96 pm, Eu^3+^=95 pm) and the pure products gave absorption spectra characteristic of TDs, and as expected the inclusion of Nd^III^ and Eu^III^ led to dramatic expansion of the chemical shift range observed in the NMR spectra. The salts of smaller ions Tb (92 pm), Dy (91 pm) and Yb (87 pm) failed to produce easily isolable TDs suggesting a cut‐off point for stability in the constrained assemblies. More detailed examination of the reactions employing Dysprosium ions verified this preliminary conclusion. The reaction between porphyrin dyad **5**, phthalocyanine and dysprosium (acac)_3_ in octanol was monitored carefully at intervals. The formation of two new (high mass) products was observed, but prolonged heating failed to give complete conversion, with unreacted porphyrin dyad **5** always present. MALDI‐MS analysis indicated that the newly formed products were the dysprosium linked TD **16** (*m*/*z=*2235) and the product from double‐decker formation between one porphyrin of the dyad with phthalocyanine (**17**, *m*/*z=*2075). This second double‐decker product could be isolated in sufficient purity to allow preliminary characterisation, but the symmetrical TD complex **16** always contained traces of the double‐decker **17**, and it was demonstrated that the TD **16** decomposed to **17** on storage and on silica. The difference in stability between one addition (double‐decker) and two (TD) is likely to be a direct consequence of the role of the bridging chain, which directs an eclipsed arrangement in the (low stability) TD. The potential for controlled sequential assembly of heterometallic TDs was demonstrated through reaction of intermediate **17** with lanthanum (acac)_3_. The La‐Dy TD **18** was indeed formed as an inseparable mixture alongside the symmetrical La‐La TD **7** (the product of TD formation and further exchange of La for Dy). Prolonged reaction with an excess of lanthanum salt led to complete exchange and formation of the La‐La TD **7** exclusively.

**Scheme 2:**
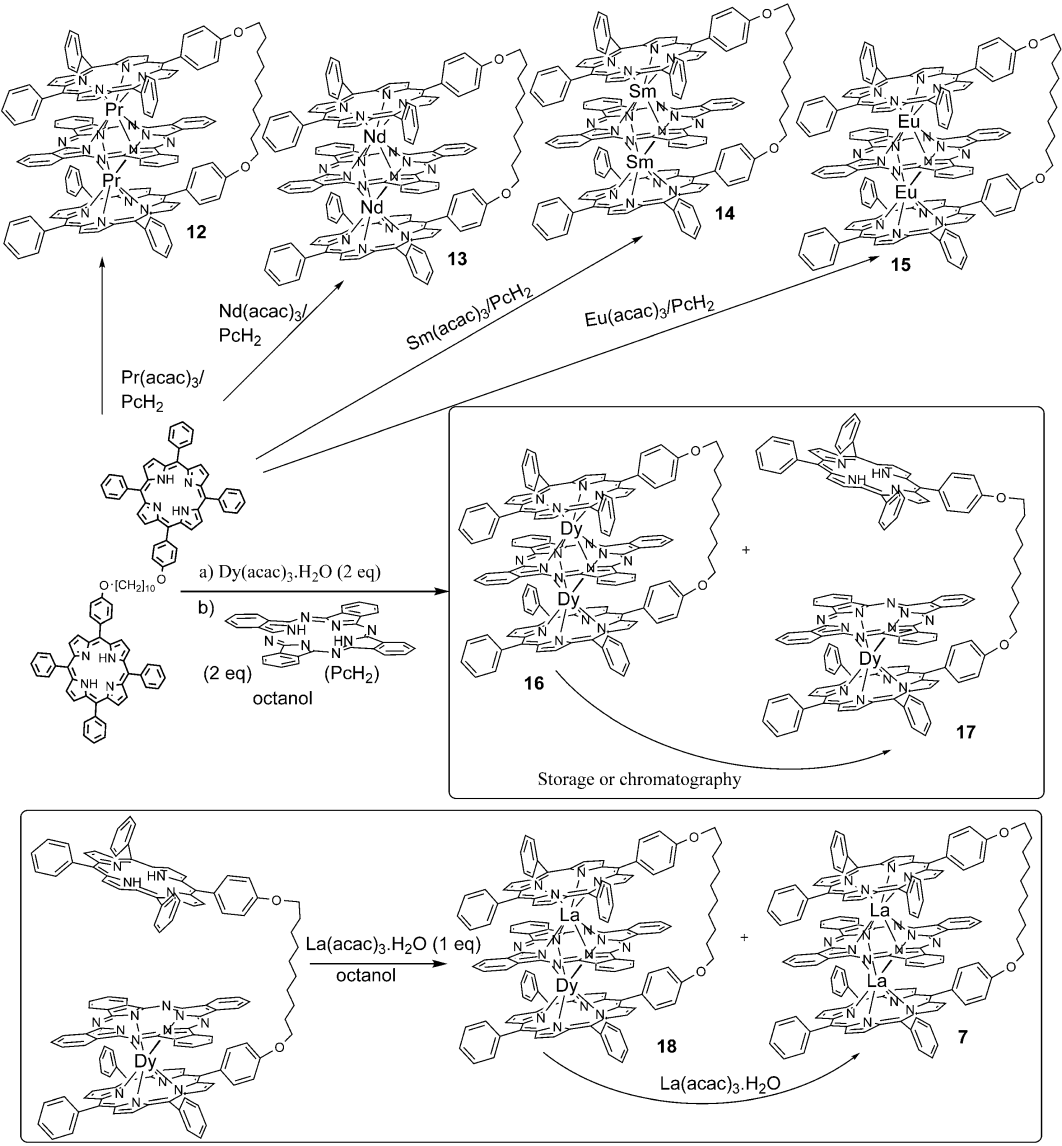
Single‐step and sequential synthesis of TD complexes using lanthanide ions of different radii.

In conclusion, a versatile and straightforward protocol is disclosed for controlled synthesis of complex lanthanide‐bridged heteroleptic porphyrin–phthalocyanine–porphyrin triple‐decker assemblies. The protocol complements the few known approaches, importantly using a simple flexible link chain between the outer components of the structure to guide the assembly of complementary, size‐matched metal ions and central macrocycle. The system displays an intricate balance of steric, geometric and ion‐size effects that introduce further control. Assembly is spontaneous as the lanthanide series is spanned from lanthanum to europium and at this point the constraints of the bridge and porphyrin substituents prevent inclusion of smaller ions. The bridging chain can be passive or active in determining the final character of the complexes—it directs the ring–ring conformation and can also prevent free rotation of the (suitably designed) central unit of the molecular gyroscope. The combination opens a new, scalable approach to robust, highly functional single‐molecule materials.

## Conflict of interest

The authors declare no conflict of interest.

## Supporting information

As a service to our authors and readers, this journal provides supporting information supplied by the authors. Such materials are peer reviewed and may be re‐organized for online delivery, but are not copy‐edited or typeset. Technical support issues arising from supporting information (other than missing files) should be addressed to the authors.

Supplementary
