# Supplementary material for: Straightforward and Controlled Synthesis of Porphyrin–Phthalocyanine–Porphyrin Heteroleptic Triple‐Decker Assemblies
Source: Chemistry. 2020 Jul 27;26(47):10724–8. doi: 10.1002/chem.202002500 (PMC7497087; doi:10.1002/chem.202002500)
Supplement: Supplementary file 1 — Supplementary [file CHEM-26-10724-s001.pdf]

Chemistry–A European Journal

Supporting Information

Straightforward and Controlled Synthesis of Porphyrin–Phthalocyanine–Porphyrin Heteroleptic Triple-Decker Assemblies

Daniel González-Lucas,[a] Shazia C. Soobrattee,[a] David L. Hughes,[a] Graham J. Tizzard,[b] Simon J. Coles,[b] and Andrew N. Cammidge*[a]

Author Contributions

D.G. Investigation: Lead
S.S. Investigation: Equal
D.H. Investigation: Supporting
G.T. Investigation: Supporting
S.C. Investigation: Supporting.

General methods:

Reagents and solvents were purchased from commercial sources and used without further purification. Rrecrystallisations were performed using distilled solvents. ^{1}H and ^{13}C NMR spectra were recorded at 500.1, and 125.7, MHz, respectively, using a Bruker AscendTM 500 spectrometer. The residual solvent peaks were used as references. Thin layer chromatography (TLC) was carried out on aluminium sheets coated with silica gel 60 F252 (Merck), with visualization by UV light. Column chromatography was carried out on silica gel Davisil® LC60A 40-63 micron (Grace GmbH & Co). MALDI-TOF mass spectra were obtained using a Shimadzu Biotech Axima instrument and isotope patterns compared to theoretical predictions. UV-Vis spectra were recorded at room temperature on a Hitachi U-3000 spectrophotometer. Melting points were measured using a Reichert Thermovar microscope with a thermopar based temperature control. X-Ray crystallography data was collected and analysed by Prof Simon J. Coles and Dr Graham J. Tizzard at the UK National Crystallography Service at Southampton and by Dr David Hughes at UEA.

Porphyrin dyad 5

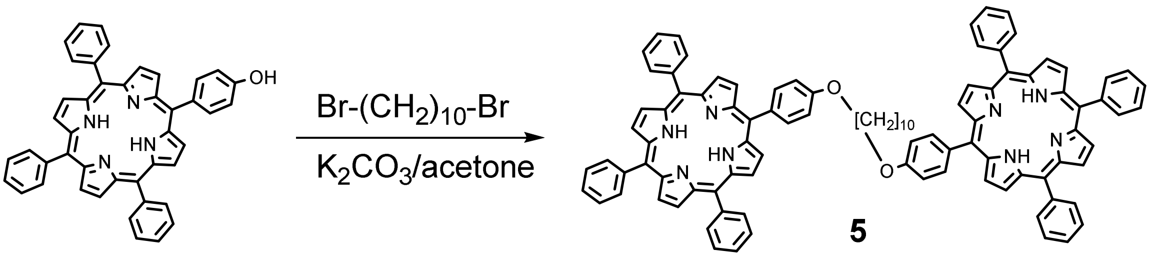

A mixture of 1,10-dibromodecane (47.6 mg, 0.16 mmol) and 5-(4-hydroxyphenyl)-10,15,20-triphenylporphyrin (200 mg, 0.32 mmol) was dissolved in acetone (40 mL), then an excess of K_2CO_3 (220 mg, 1.5 mmol) was added and the mixture heated at reflux for 48h. The solution was decanted and MeOH added to precipate the porphyrin dyad which was recrystallised twice from DCM:MeOH to yield the pure product **5** as a purple solid (127 mg, 57.3 %yield). m.p. > 350 °C. ^{1}H NMR (500 MHz, $CDCl_3$): δ 8.89 (d, J = 4.5 Hz, 4H) and 8.83 (d, J = 4.5 Hz, 12H) H_β; 8.21 (dd, J = 7, <2 Hz, 12H) H_{oPh}; 8.12 (dd, J = 7, <2 Hz, 4H) $H_{oPh'}$; 7.79 – 7.70 (m, 18H) H_{mPh} and H_{pPh}; 7.29 (dd, J = 7, <2 Hz, 4H) $H_{mPh'}$; 4.28 (t, J = 6.5 Hz, 4H) O-CH_2-CH$_2$-; 2.07 – 1.99 (m, 4H) O-CH$_2$-CH_2-; 1.72 – 1.65 (m, 4H) -CH_2-; 1.53 (s, 8H) -CH_2-; -2.76 (s, 4H) NH. ^{13}C NMR (500 MHz, $CDCl_3$): δ 159.18, 142.38, 135.77, 134.70, 134.48, 127.82, 126.81, 125.68, 120.35, 120.20, 120.07, 112.90, 77.41, 77.16, 76.91, 68.51, 30.48, 29.83, 29.74, 26.45. MS (MALDI-tof): m/z = 1399.61 [M$^+$]. UV-vis, (DCM)/nm (log ε): 418(5.3), 514(3.9), 551(3.7), 593(3.5), 649(3.7). IR (KBr, cm^{-1}):2928, 2858, 1600, 1514, 1471, 1442, 1349, 1246, 965.

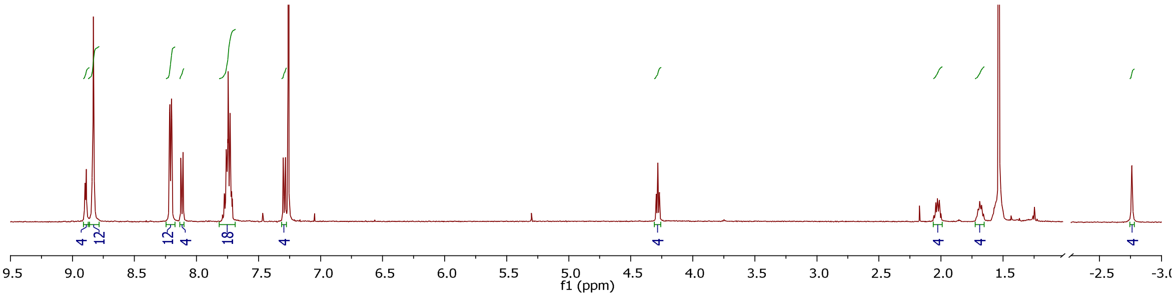

Lanthanum triple decker 7

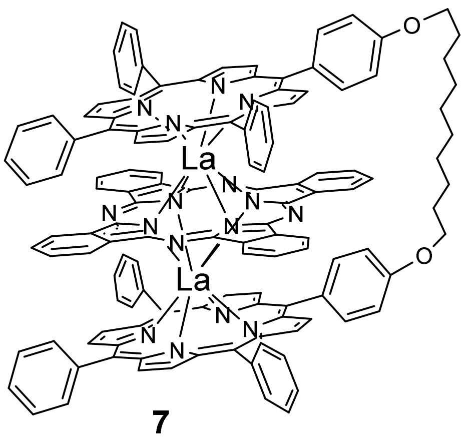

7

Porphyrin dyad **5** (200 mg, 0.143 mmol) was mixed with lanthanum(III) acetylacetonate hydrate (125 mg, 0.286 mmol) and dissolved in octanol (15 mL). The mixture was heated at reflux under Ar for 16 h. An aliquot was removed and analysed by uv-vis spectroscopy to check metallation was complete. Then, phthalocyanine (73 mg, 0.143 mmol) was added and the mixture was heated at reflux for 18h under Ar. The solvent was removed by distillation under reduced pressure and the crude residue recrystallised from DCM:MeOH. The resulting solids were then separated by column chromatography through silica gel using DCM:pet ether (6:4 v/v) as eluent, and recrystallized rom DCM:MeOH to give the lanthanum triple decker **7** as a dark brown solid (268 mg, 86 %). ^{1}H NMR (500 MHz, $CDCl_3$) δ 10.08 (d, *J* = 7.2 Hz, 2H) $H_{oiPh'}$; 9.99 (t, *J* = 7 Hz, 6H) H_{oiPh}; 9.36 (dd, *J* = 5, 3 Hz, 8H) H_{pC}; 8.48 – 8.40 (m, 6H) H_{ooPh}; 8.29 (dd, *J* = 5, 3 Hz, 8H) H_{pC}; 7.98 (d, *J* = 6.5 Hz, 2H) $H_{ooPh'}$; 7.86 – 7.77 (m, 6H) H_{miPh}; 7.31 (d, *J* = 4 Hz, 4H) H_{β}; 7.26 – 7.21 (m, 18H) H_{β}, H_{miPh} and H_{pPh} ; 6.87 (d, *J* = 6.0 Hz, 2H) $H_{miPh'}$; 6.73 (d, *J* = 7Hz, 2H) $H_{moPh'}$; 6.64 (t, *J* = 7 Hz, 6H) H_{moPh}; 4.59 (t, *J* = 7 Hz, 4H) -O-CH_2- ; 2.33 – 2.23 (m, 4H) -CH_2-; 1.92 (m, 8H) -CH_2-; 1.81 (s, 4H) -CH_2-. ^{13}C NMR (126 MHz, $CDCl_3$) δ 158.74, 153.70, 148.23, 147.88, 143.14, 136.74, 133.57, 133.43, 130.19, 128.57, 128.46, 127.74, 127.25, 125.97, 125.93, 123.60, 120.22, 120.12, 68.37, 29.62, 29.39, 28.75, 25.96. MS (MALDI-tof): m/z = 2186.05 (cluster) [M^+]. UV-vis, (DCM)/nm(log ε): 360(4.8), 419(5.2), 485(2.7), 550(2.5), 605(2.6). IR (KBr, cm^{-1}): 3053, 2928, 2855, 1606, 1513, 1469, 1439, 1406, 1330, 1288, 1243, 1198, 1177, 1116, 1003, 984, 880.

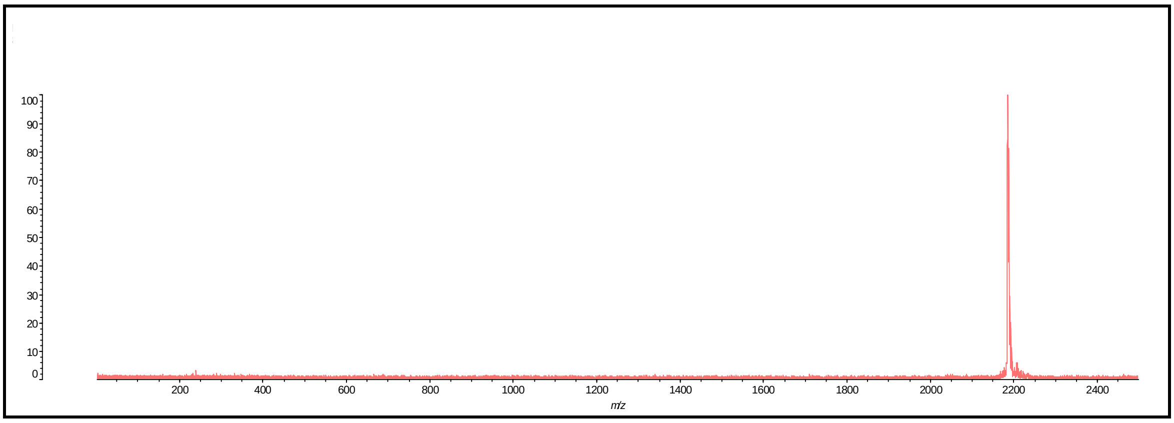
m/z

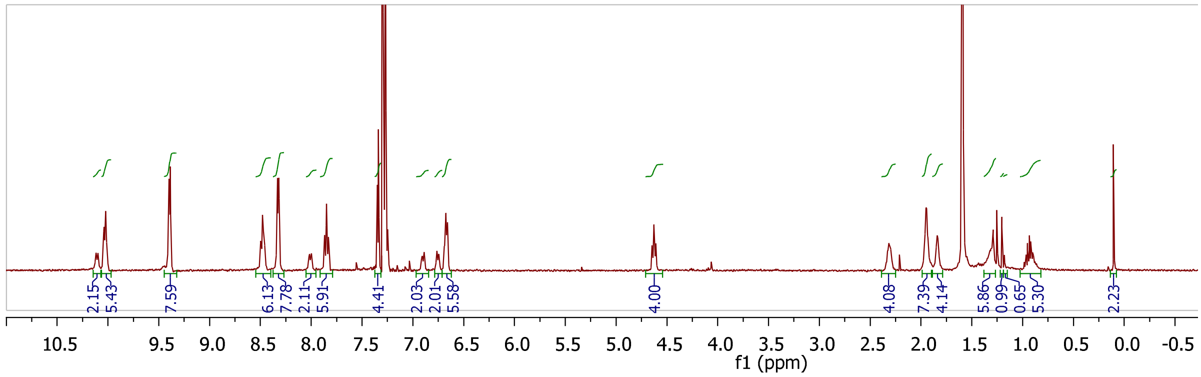
f1 (ppm)

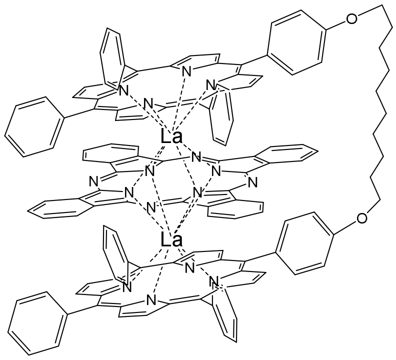

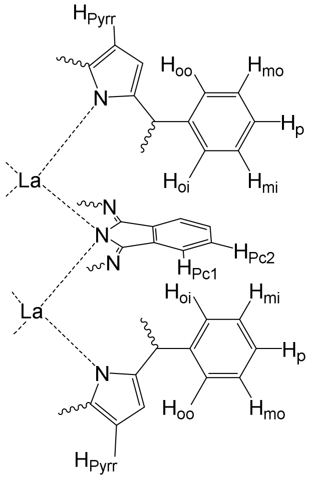
HPyrr
Hoo
Hmo
Hp
Hoi
Hmi
La
N
HPc2
HPc1

7

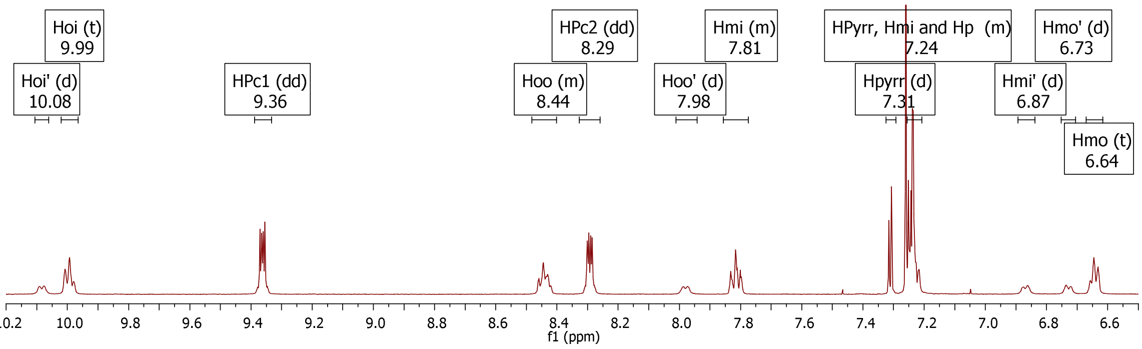
Hoi (t)
9.99
Hoi' (d)
10.08
HPc1 (dd)
9.36
HPc2 (dd)
8.29
Hoo (m)
8.44
Hmi (m)
7.81
Hoo' (d)
7.98
HPyrr, Hmi and Hp (m)
7.24
Hpyrr (d)
7.31
Hmo' (d)
6.73
Hmi' (d)
6.87
Hmo (t)
6.64
f1 (ppm)

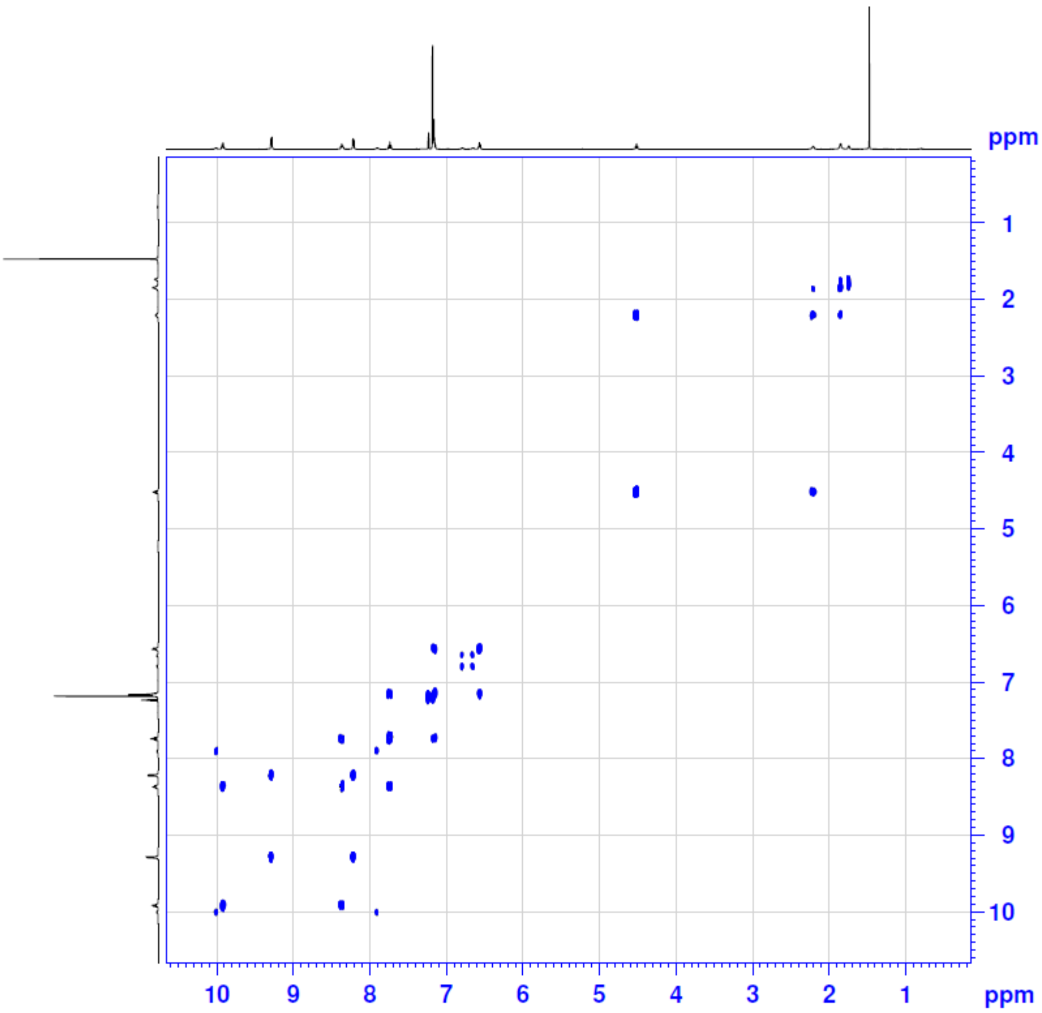
ppm
1
2
3
4
5
6
7
8
9
10
10
9
8
7
6
5
4
3
2
1
ppm

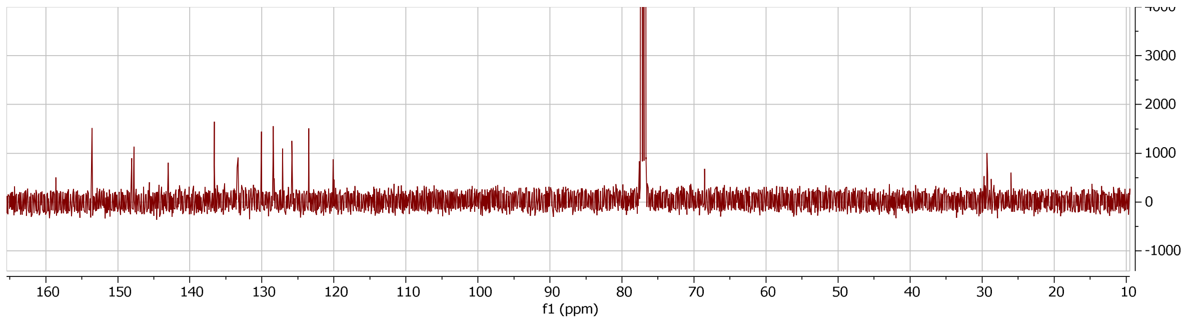
3000
2000
1000
0
-1000
160
150
140
130
120
110
100
90
80
70
60
50
40
30
20
10
f1 (ppm)

Bis-triple decker 6

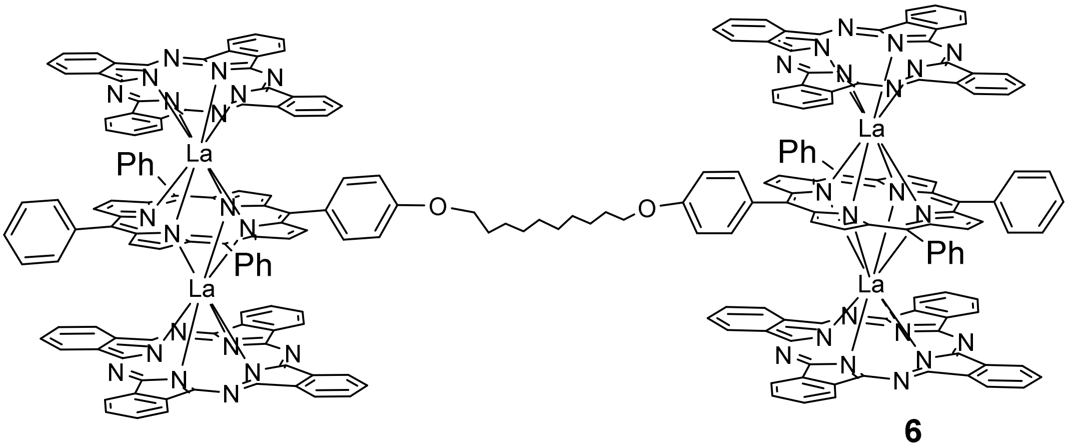

6

Porphyrin dyad **5** (25 mg, $17.9x10^{-3}$ mmol) was mixed with lanthanum(III) acetylacetonate hydrate (32.7 mg, $76.0x10^{-3}$ mmol) and dissolved in octanol (10 mL). The mixture was heated at reflux under Ar overnight and then an excess of phthalocyanine (92 mg, $179.0x10^{-3}$ mmol) was added. The mixture was heated at reflux under Ar for 16 h then cooled. Pet. Ether was added and the resulting green solid was filtered off and further purified by column chromatography through silica gel using DCM as eluent. The green fraction was concentrated to give bis-triple decker **6** as a dark green solid (24.9 mg, 34 %).
^{1}H NMR (400 MHz, $CDCl_3$) δ 10.01 (d, J = 7.0 Hz, 12H) H_{Arpor}; 9.91 (d, J = 8.0 Hz, 5H) H_{Arpor}; 8.82 (dd, J = 5.5, 3 Hz) H_{ArpC}; 8.41 (t, J = 7.5 Hz, 13H) H_{Arpor}; 8.23 – 8.14 (m, 9H) H_{Arpor}; 8.03 – 7.93 (m, 9H) H_{Arpor}; 7.84 (dd, J = 5.5, 3 Hz, 32H) H_{ArpC}; 7.76 (d, J = 4.4 Hz, 3H) H_{Arpor}; 7.70 (d, J = 7.8 Hz, 14H) H_{Arpor}; 4.81 (t, J = 6.5 Hz, 4H) -O-CH_2-; 2.45 (m, 4H) -CH_2-; 2.10 (m, 4H) -CH_2-, 1.93 (m, 4H) -CH_2-; 1.83 (m, 8H) -CH_2-. MS (MALDI-tof): m/z = 4005 [M^+]. UV-vis, (DCM)/nm(log ε): 347(5.4), 417(2.9), 669(4.6), 705(4.7). IR (KBr, cm^{-1}): 2961, 2917, 2849, 1645, 1463, 1261, 1093, 1020, 879. (A complete ^{13}C NMR spectrum could not be obtained for this compound due to the low solubility).

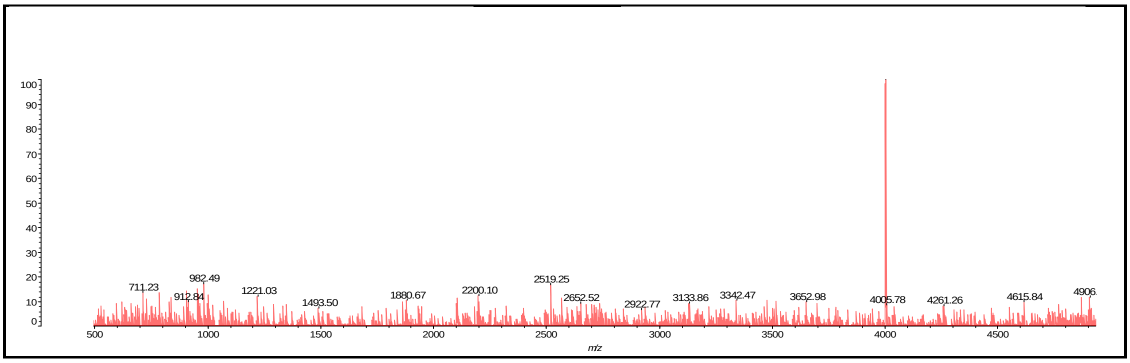

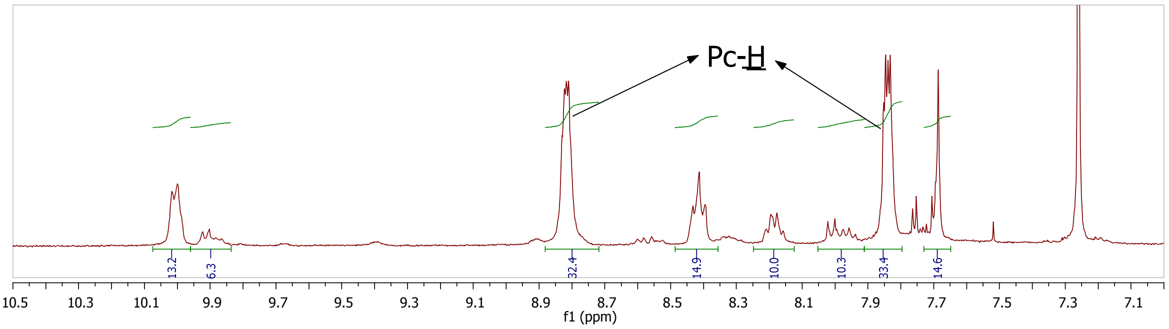

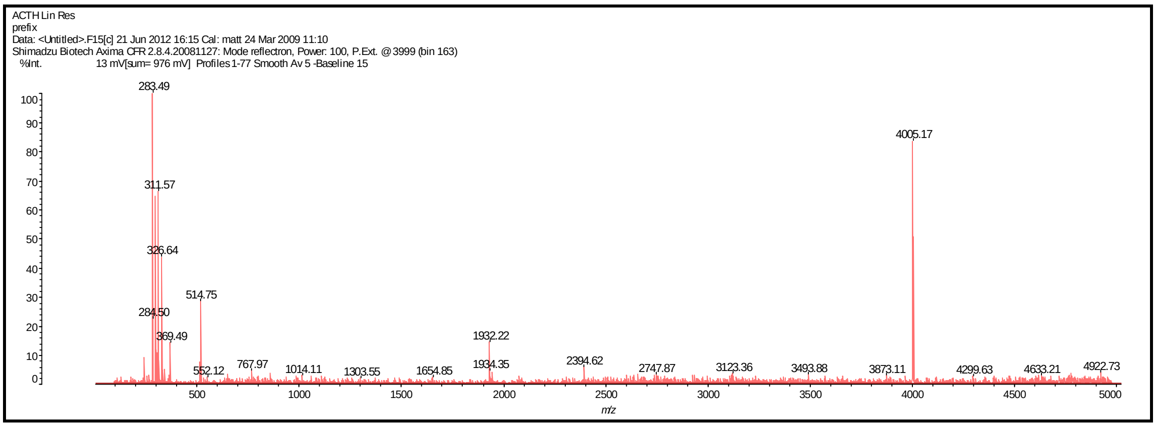
ACTH Lin Res
prefix
Data: <Untitled>.F15[c] 21 Jun 2012 16:15 Cal: matt 24 Mar 2009 11:10
Shimadzu Biotech Axima CFR 2.8.4.20081127: Mode reflectron, Power: 100, P.Ext. @ 3999 (bin 163)
%Int.
13 mV[sum= 976 mV] Profiles 1-77 Smooth Av 5 -Baseline 15
283.49
4005.17
311.57
326.64
514.75
284.50
369.49
1932.22
1934.35
2394.62
552.12
767.97
1014.11
1303.55
1654.85
2747.87
3123.36
3493.88
3873.11
4299.63
4633.21
4922.73
m/z

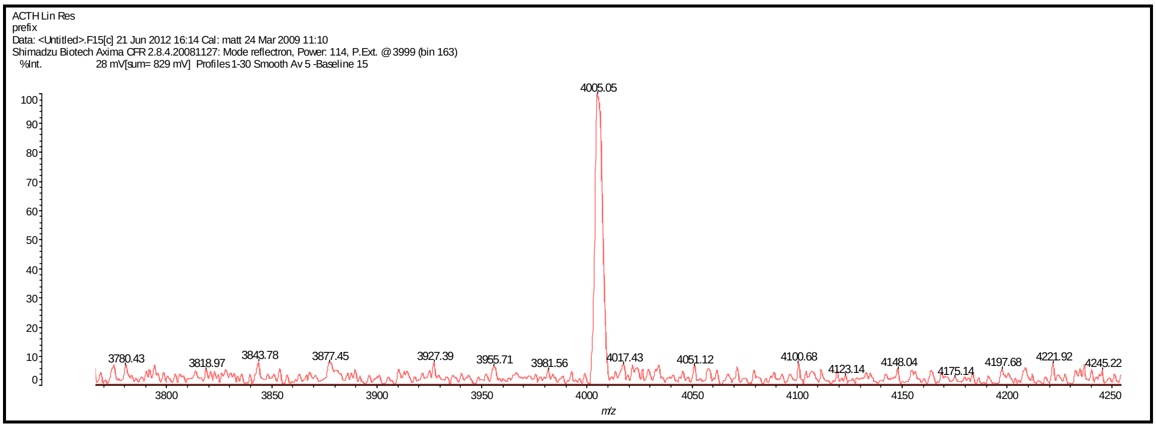
ACTH Lin Res
prefix
Data: <Untitled>.F15[c] 21 Jun 2012 16:14 Cal: matt 24 Mar 2009 11:10
Shimadzu Biotech Axima CFR 2.8.4.20081127: Mode reflectron, Power: 114, P.Ext. @ 3999 (bin 163)
%Int.
28 mV[sum= 829 mV] Profiles 1-30 Smooth Av 5 -Baseline 15
4005.05
3780.43
3818.97
3843.78
3877.45
3927.39
3955.71
3981.56
4017.43
4051.12
4100.68
4123.14
4148.04
4175.14
4197.68
4221.92
4245.22
m/z

Neodymium triple decker 13 (General procedure)

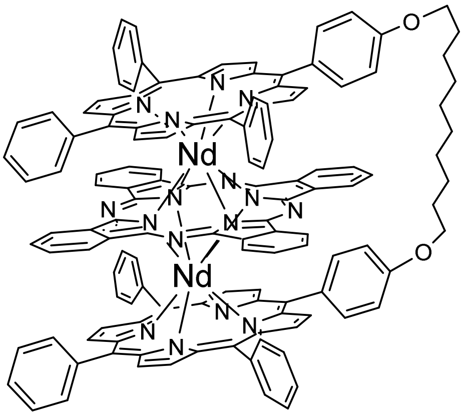

13

Porphyrin dyad **5** (60 mg, 0.043 mmol), Nd $(acac)_3.H_2O$ (37.9 mg, 0.086 mmol), phthalocyanine (22.0 mg, 0.043 mmol) were heated in refluxing octanol (10 ml) for 7h. The reaction was allowed to cool, methanol added, and the mixture left to precipitate overnight. The solid was filtered off and further purified by column chromatography through silica gel using DCM:hexane as eluent. The green fraction was concentrated to give neodymium triple decker **13** as a dark green solid (20.1 mg, 21 %). ^{1}H NMR NMR (500 MHz, Acetone) δ 8.38 (d, *J* = 7.6 Hz, 2H), 8.33 (d, *J* = 7.4 Hz, 4H), 8.02 (d, *J* = 8.2 Hz, 2H), 7.45-7.27 (m, 20H), 6.97 – 6.90 (m, 6H), 6.83 (d, *J* = 6.8 Hz, 2H), 6.45-6.38 (m, 6H), 6.07 (br s, 2H), 5.35 (br s, 8H), 4.62 – 4.38 (m, 16H), 3.66 (t, *J* = 7.5 Hz, 4H), 1.64 – 1.55 (m, 4H), 1.27 – 1.08 (m, 8H), 0.87 (br t, *J* = 7.5 Hz, 4H); ^{13}C NMR (126 MHz, $CDCl_3$) δ 157.5, 152.2, 152.0, 151.8, 140.2, 136.7, 136.3, 134.1, 132.2, 127.8, 126.2, 125.7, 124.4, 117.1, 113.3, 67.9, 29.8, 28.9, 28.6, 27.9, 26.0; MS (MALDI-tof): m/z = 2196.65 [M+]; IR (ATR, solid film cm^{-1}): 3050, 2918, 2855, 1601, 1513, 1472, 1406, 1326, 1238, 1198, 1174, 1116, 984, 880; UV-vis, (DCM)/nm: 361, 420, 488, 550, 610.

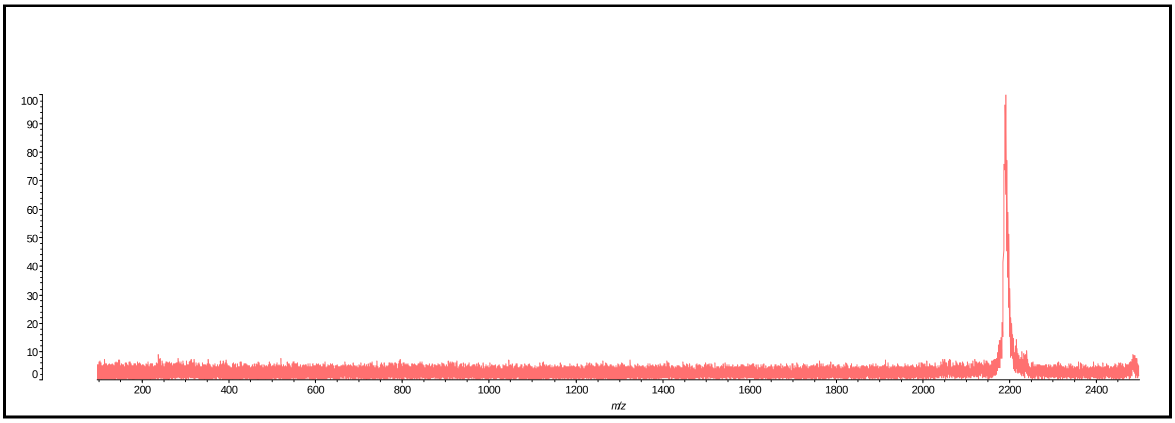

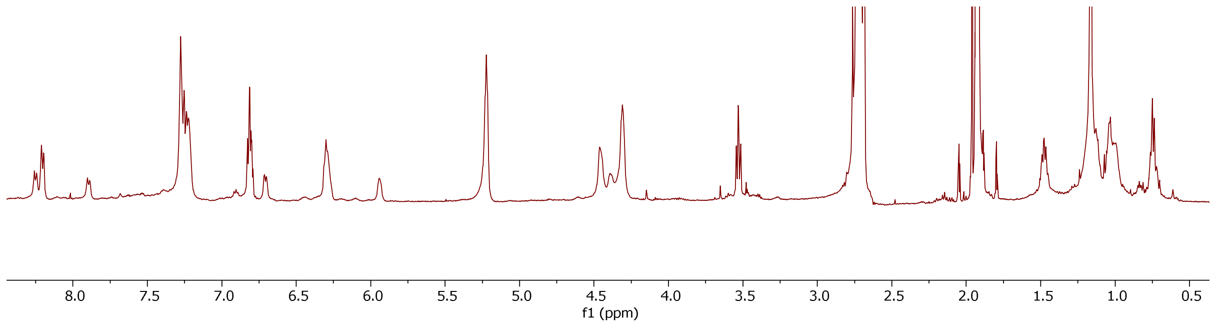
8.0
7.5
7.0
6.5
6.0
5.5
5.0
4.5
4.0
3.5
3.0
2.5
2.0
1.5
1.0
0.5
f1 (ppm)

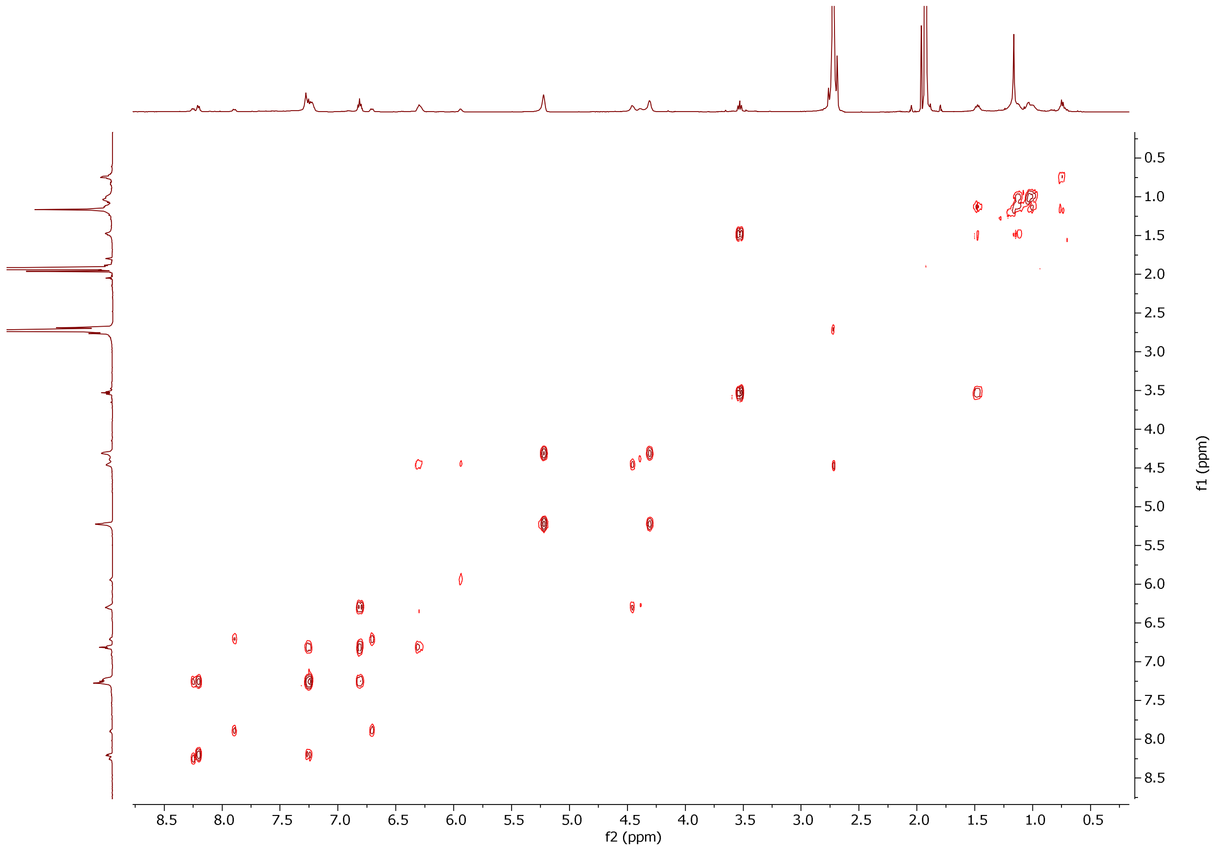
8.5
8.0
7.5
7.0
6.5
6.0
5.5
5.0
4.5
4.0
3.5
3.0
2.5
2.0
1.5
1.0
0.5
f2 (ppm)
f1 (ppm)

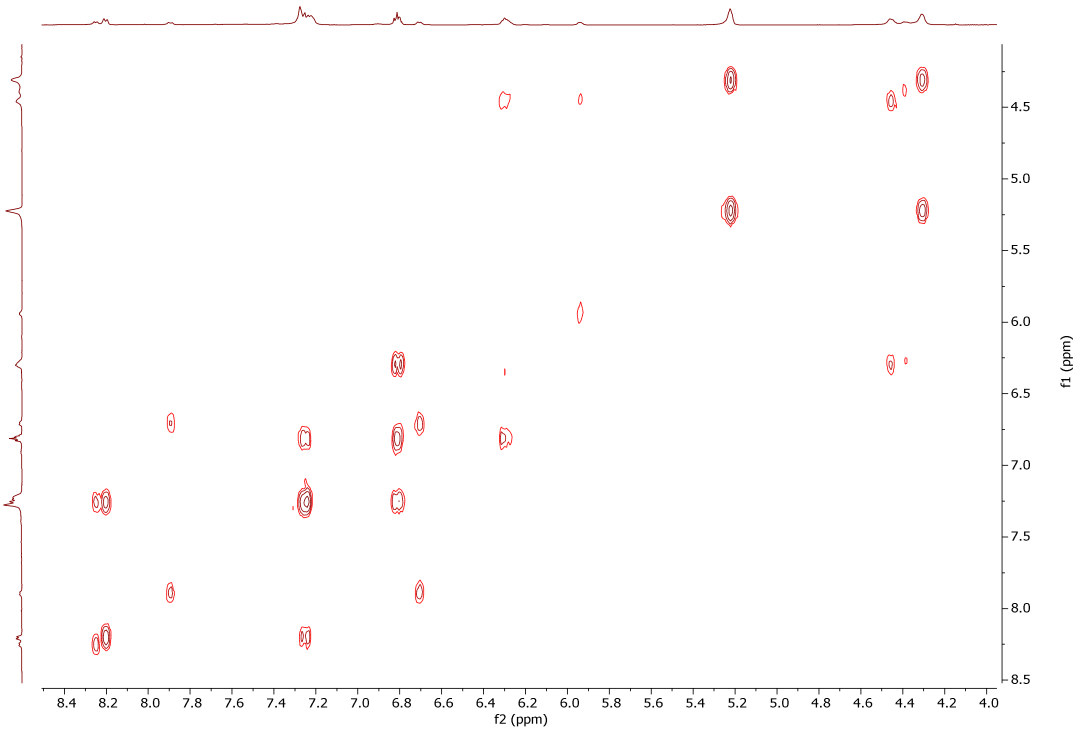
f2 (ppm)
f1 (ppm)

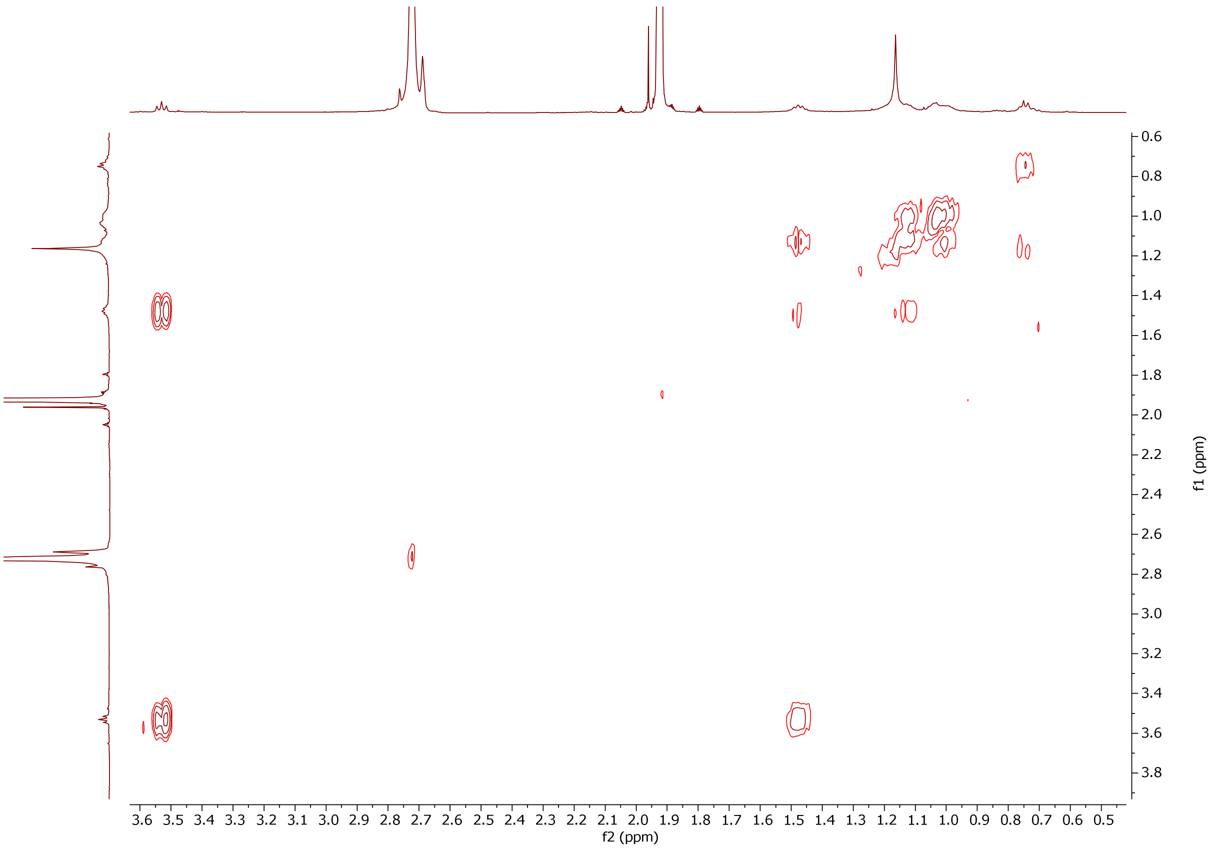
f2 (ppm)
f1 (ppm)

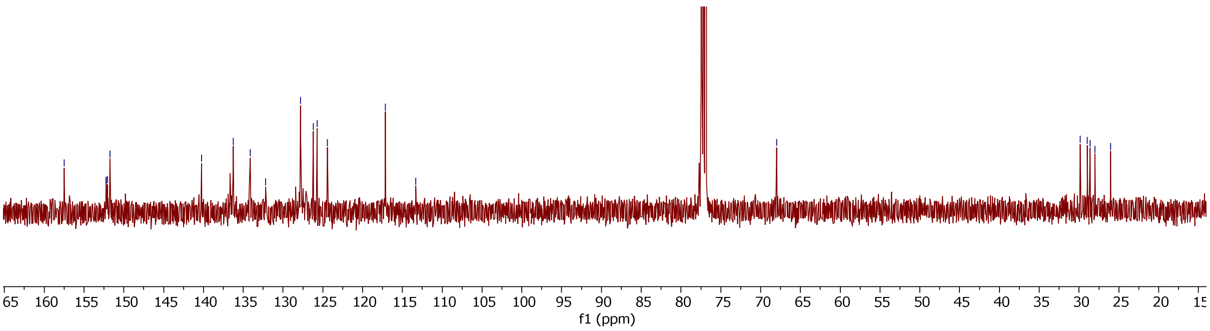

Praseodymium triple decker 12 (prepared following the general procedure described for 13)

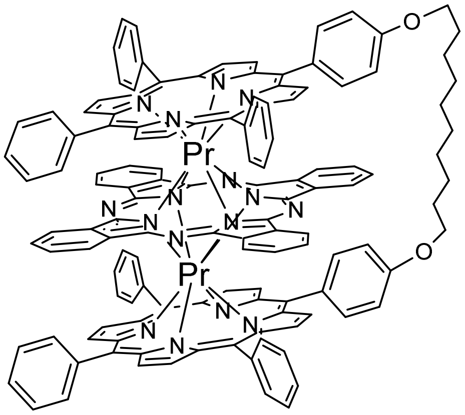

^{1}H NMR NMR (500 MHz, $CDCl_3$) δ 9.34 (d, *J* = 8.8 Hz, 2H), 9.30 (d, *J* = 8.0 Hz, 4H), 8.74 (d, *J* = 8.0 Hz, 2H), 7.32 (t, *J* = 7.0 Hz, 6H), 6.68 (d, J = 8.0 H, 2H), 6.43-6.36 (m, 6H), 5.81 (br s, 8H), 5.71 (br s, 4H), 5.66 (br s, 4H), 5.11-5.00 (m, 6H), 4.46-4.41 (br d, 2H), 3.91 (br s, 8H), 3.09 (t, J = 8.0 Hz, 4H), 1.70 (br s, 8H), 1.18-1.14 (m, 4H, obscured by solvent), 0.79-0.76 (m, 4H, obscured by solvent), 0.60-0.57 (m, 4H, obscured by solvent).

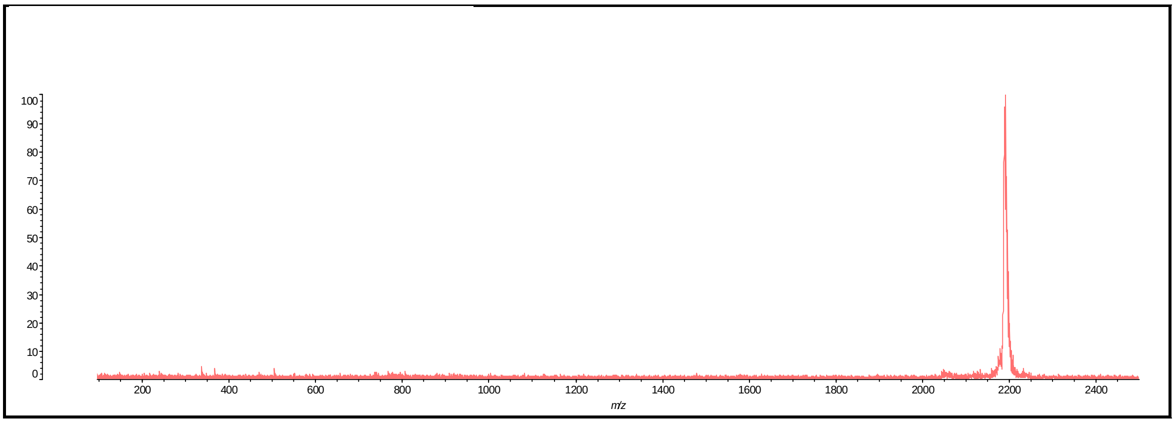

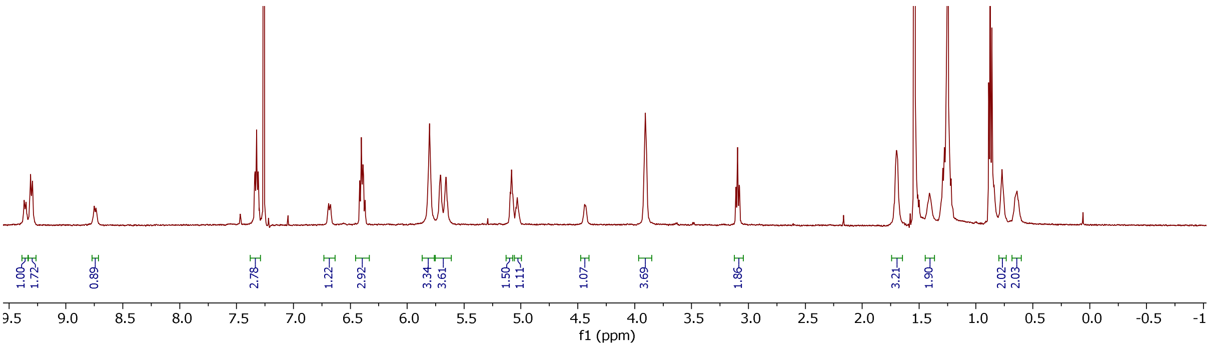

1.00
1.72
0.89
2.78
1.22
2.92
3.34
3.61
1.50
1.11
1.07
3.69
1.86
3.21
1.90
2.02
2.03
f1 (ppm)

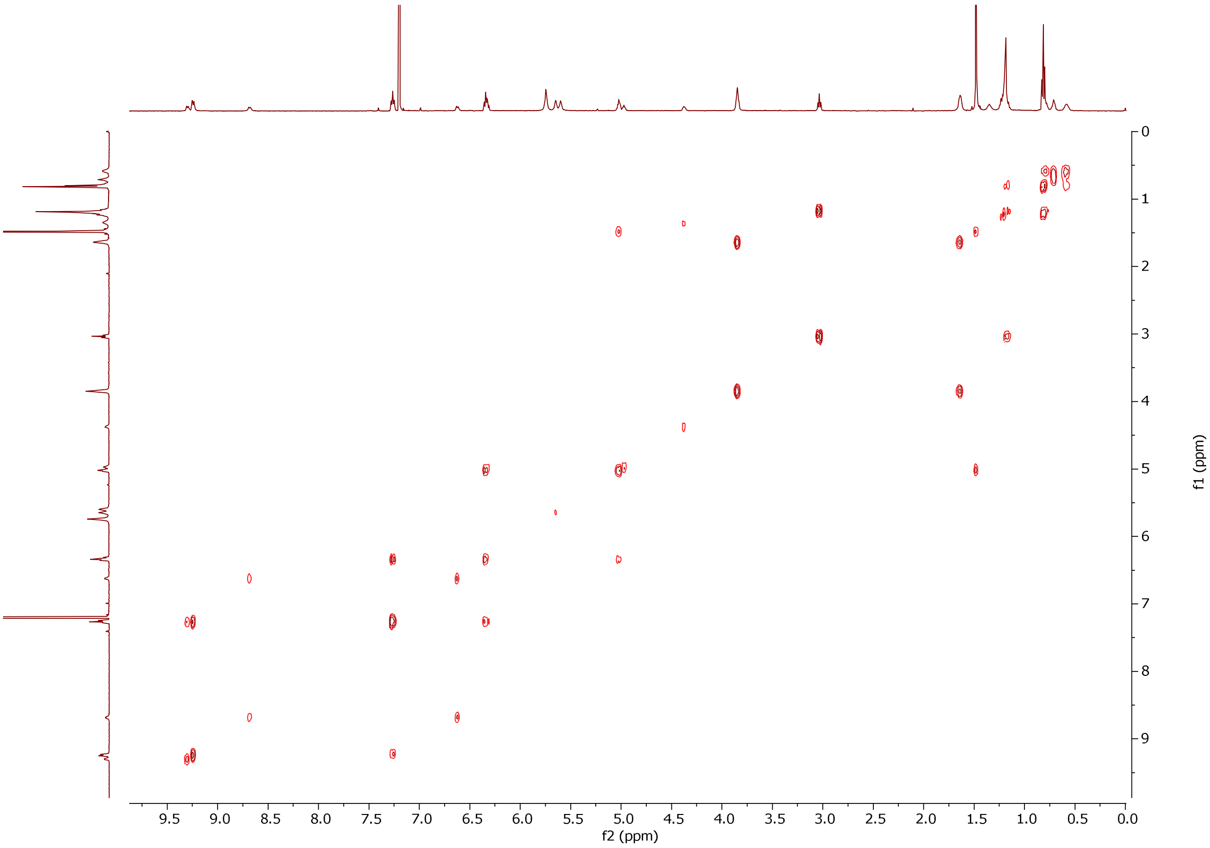

f2 (ppm)
f1 (ppm)

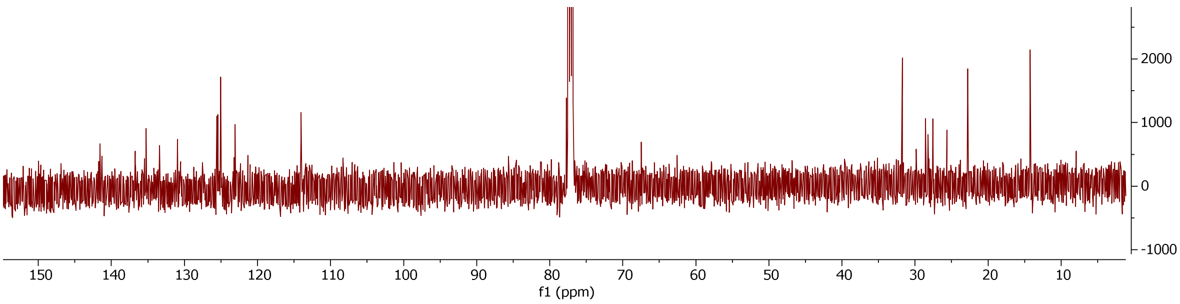

f1 (ppm)

Samarium triple decker 14 (prepared following the general procedure described for 13)

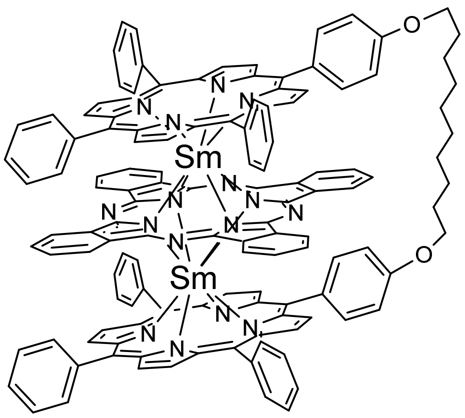

^{1}H NMR NMR (500 MHz, $CDCl_3$) δ 8.38 (d, *J* = 8.0 Hz, 6H), 8.33 (d, *J* = 8.0 Hz, 2H), 8.08-8.04 (m, 8H), 7.81-7.73 (m, 6H), 7.61-7.56 (m, 8H), 7.55-7.48 (m, 6H), 7.20-7.14 (m, 6H), 6.96-6.89 (m, 8H), 6.77 (br d, 2H), 6.51-6.40 (m, 16H), 4.32 (t, J = 8.0 Hz, 4H), 2.10-2.03 (m, 4H), 1.75-1.59 (br m, 12H); ^{13}C NMR (126 MHz, $CDCl_3$) δ 158.0, 156.4, 150.6, 150.3, 142.8, 134.8, 133.2, 132.9, 131.2, 131.1, 128.4, 125.7, 125.2, 124.5, 124.4, 124.3, 122.2, 117.7, 68.4, 29.7, 29.5, 28.9, 28.7, 26.3.

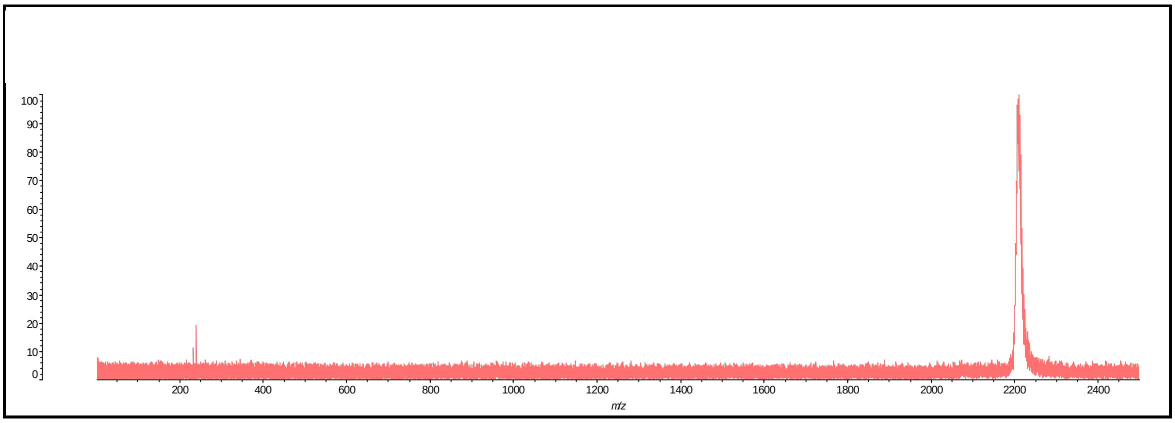

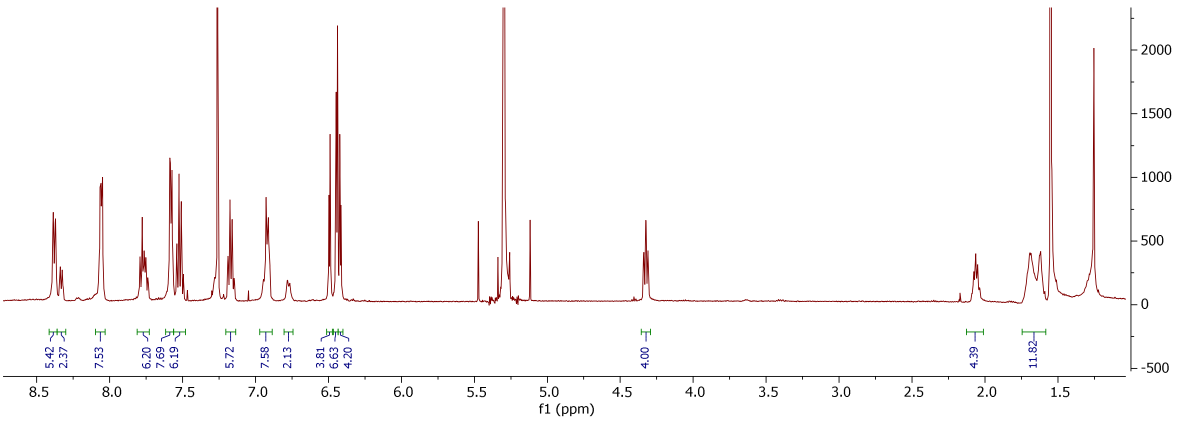

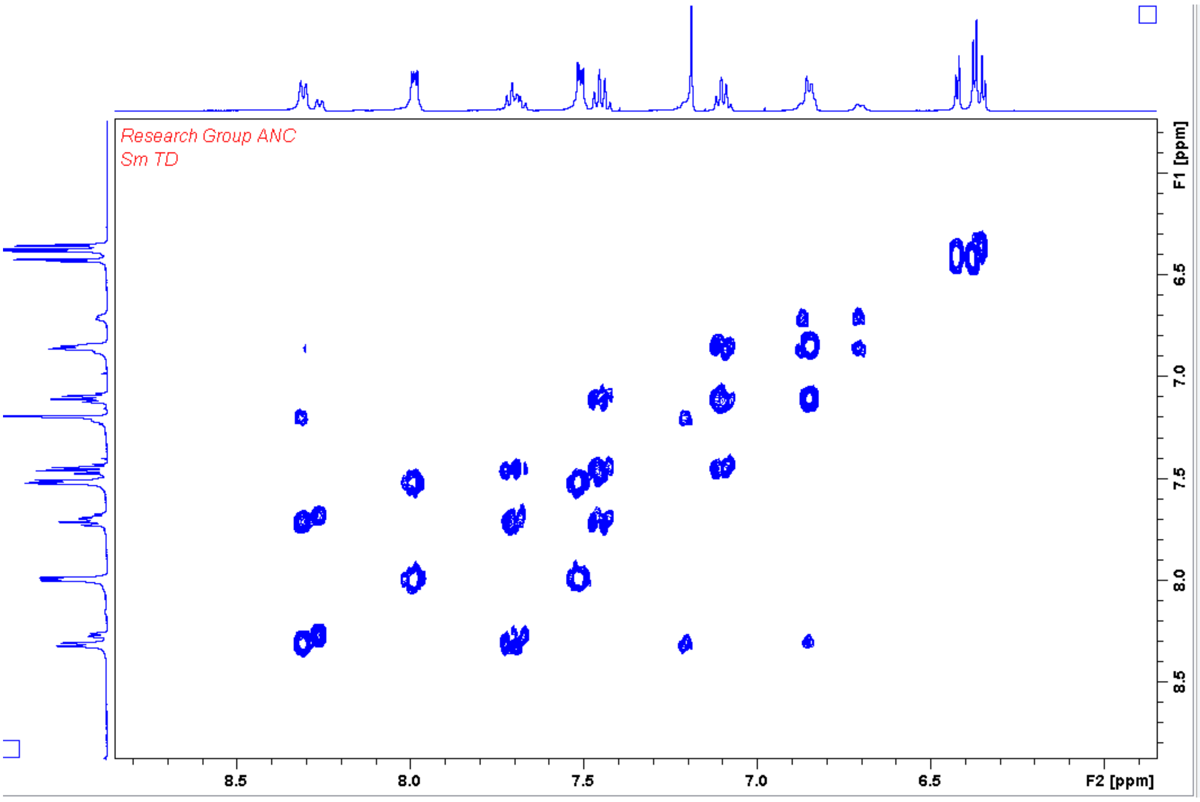

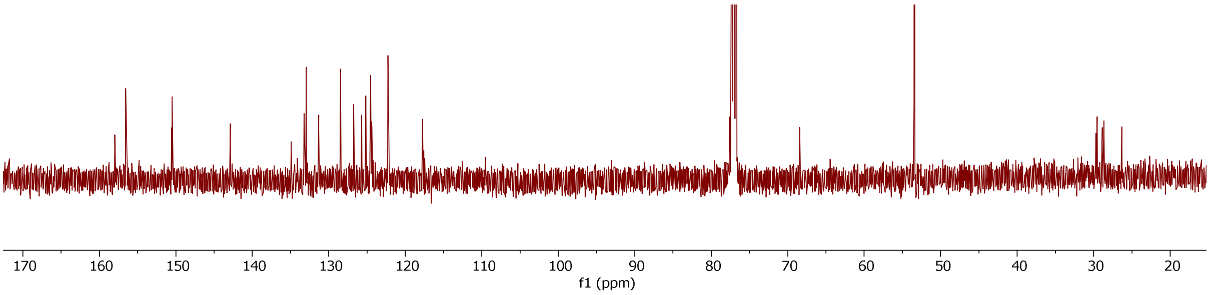

Europium triple decker 15 (prepared following the general procedure described for 13)

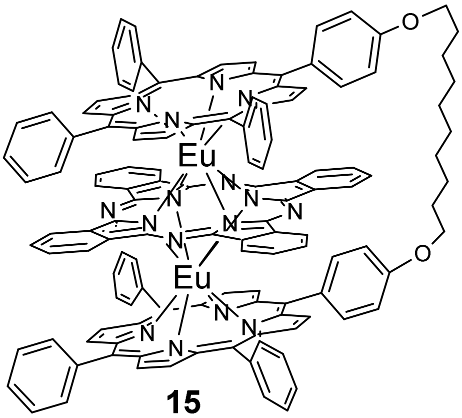

15

^{1}H NMR NMR (500 MHz, $CDCl_3$) δ 13.17 (br s, 4H), 13.00 (br s, 4H), 12.85 (br s, 8H), 10.66 (br s, 8H), 9.58-9.44 (m, 6H), 9.17 (br s, 2H), 8.40 – 8.22 (m, 6H), 6.99-6.88 (m, 6H), 6.77 (br s, 2H), 5.68 (br s, 2H), 5.21-5.06 (m 10H), 4.21 (s, 4H), 4.15 – 3.86 (m, 12H), 2.84 – 2.70 (m, 4H), 2.55 – 2.29 (m, 12H); ^{13}C NMR (126 MHz, $CDCl_3$) δ 158.8, 153.4, 130.2, 129.2, 127.8, 126.8, 125.9, 125.6, 118.3, 77.7, 74.0, 73.9, 73.7, 69.3, 53.6, 30.6, 29.9, 29.6, 27.1; MS (MALDI-tof): m/z = 2214.28 [M+]; IR (ATR, solid film cm$_{-1}$): 3052, 2920, 2850, 1602, 1513, 1472, 1406, 1326, 1288, 1238, 1198, 1174, 1116, 984, 880; UV-vis, (DCM)/nm: 360, 420, 486, 550, 605.

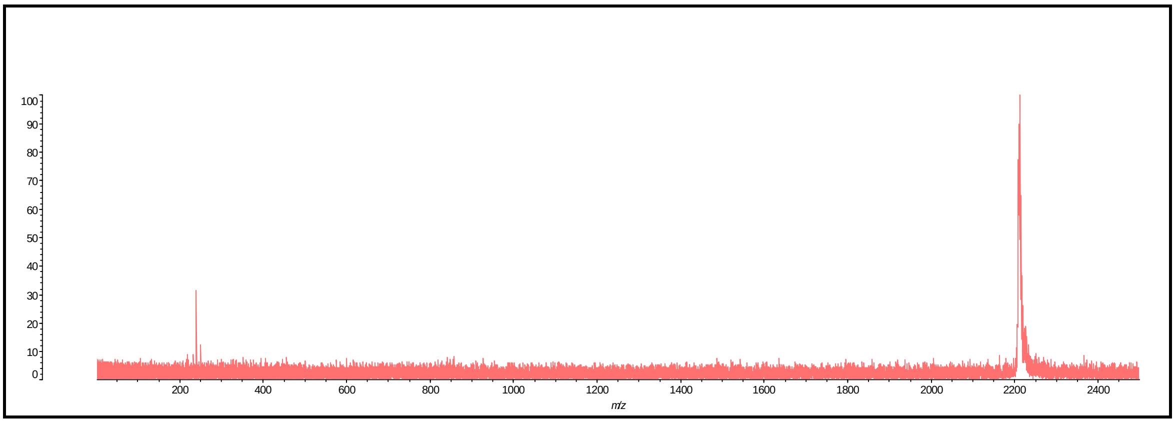

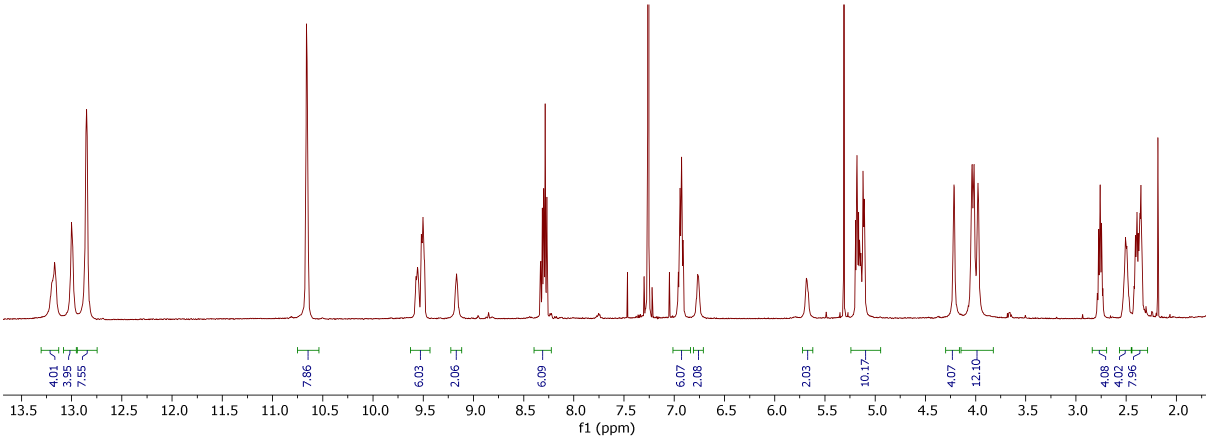

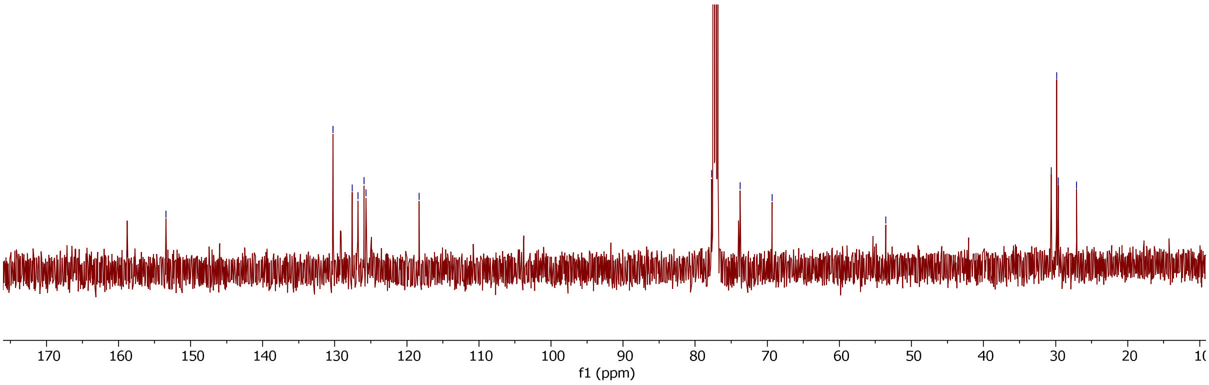

Dysprosium double decker 17

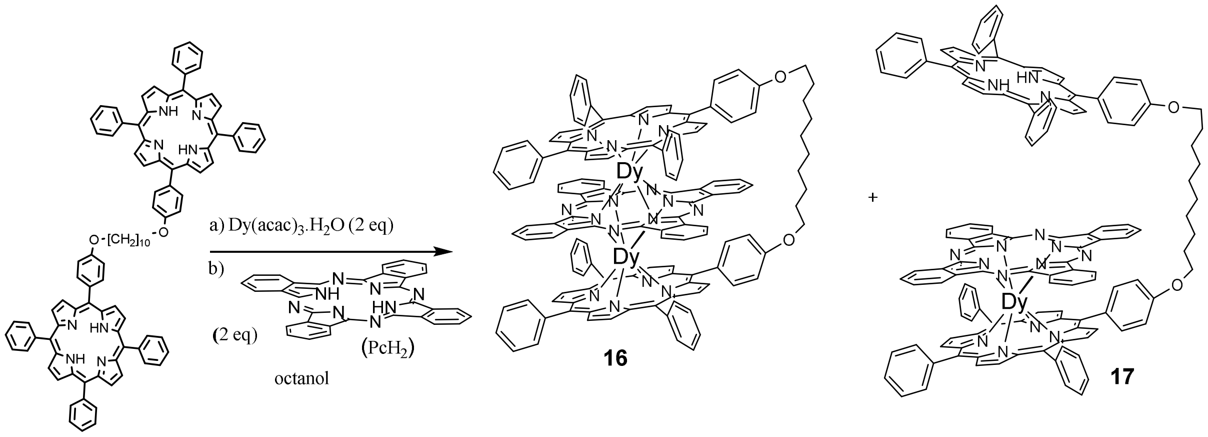

Porphyrin dyad **5** (101 mg, 0.072 mmol), $Dy(acac)_3{\cdot}H_2O$ (49.8 mg, 0.11 mmol) and PcH_2 (37.1 mg, 0.072 mmol), were dissolved in 7 ml of octanol and refluxed for 24 h. Then, the solvent was distilled under reduced pressure to obtain a dark solid that was purified by silica gel column chromatography (twice) using THF/Pet ether using EtOAc/pet. ether (1:1) as eluent to isolate a mixture of **16** and **17** (by MALDI-MS) and then using DCM/Pet ether (3:2). The resultant purple-brown solid was then recrystallised from DCM/MeOH to yield the pure product **17** (51 mg, 34 %). ^1H NMR (500 MHz, CD_2Cl_2) δ 9.08 (br d, 2H), 8.95 (br d, 2H), 8.86 (br d, 1H), 8.82-8.74 (br m, 3H), 8.83-8.65 (br m, 2H), 8.64-8.57 (br m, 2H), 8.34 (br d, 2H), 8.19-8.08 (m, 3H), 7.93-7.83 (m, 2H), 7.77-7.66 (m, 3H), 7.45 (br d, 2H), 7.30-7.26 (m, 1H), 6.28 (br d, 2H), 4.28-4.21 (m, 2H), 2.88 (br t, 2H), 1.22 (m, obscured by solvent), 0.24 ((br pe, 2H), -0.37 (br pe, 2H), -0.87 (br pe, 2H), -1.35 (br pe, 2H), -1.89 (br pe, 2H), -2.46—2.56 (br m, 2H), -2.91 (br d, 2H), -3.46- -3.64 (br m, 4H), -3.88 (br s, 2H), -4.08 (br s, 2H), -19.00 – -19.15 (br m, 4H), -31.88 (br s, 4H), -54.74 (br s, 3H), -65.62 (br s, 1H), -70.64 (br s, 2H). MS (MALDI-tof): m/z = 2076.06 [M^+]. (A complete ^{13}C NMR could not be obtained).

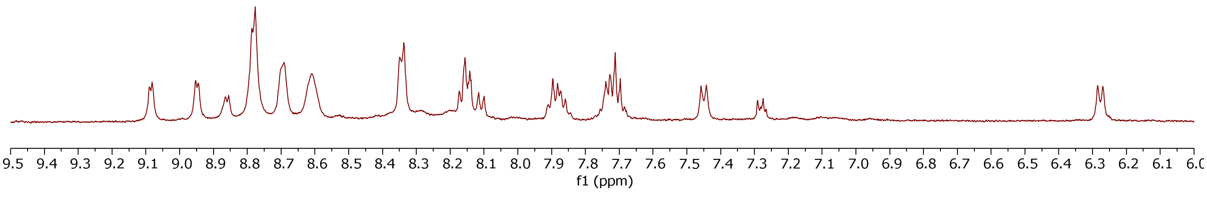

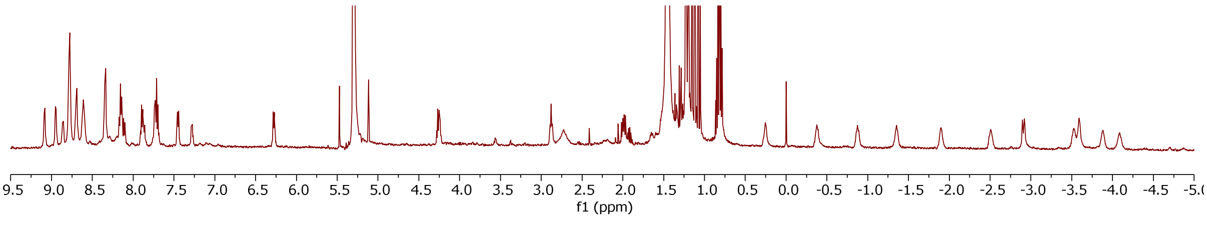

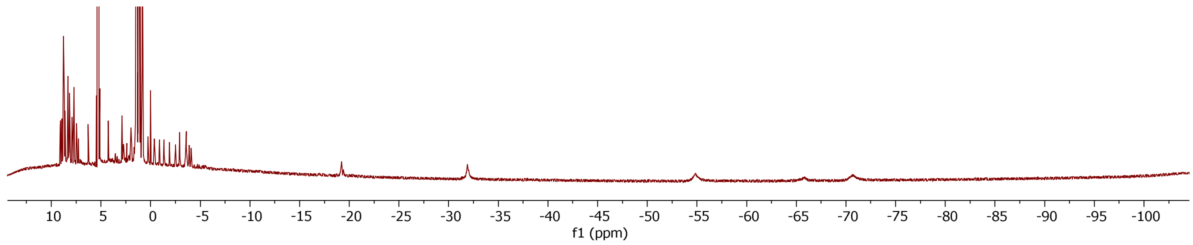

10 5 0 -5 -10 -15 -20 -25 -30 -35 -40 -45 -50 -55 -60 -65 -70 -75 -80 -85 -90 -95 -100
f1 (ppm)

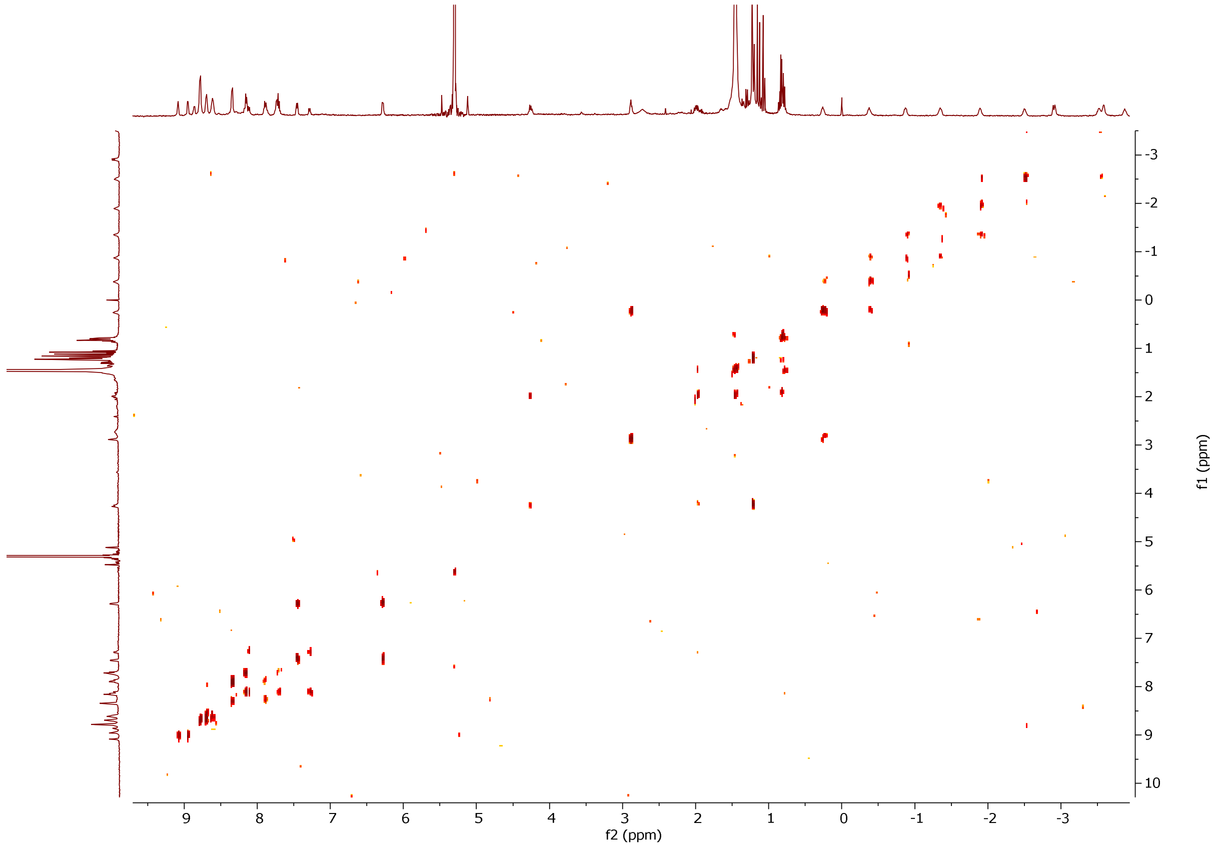

9 8 7 6 5 4 3 2 1 0 -1 -2 -3
f2 (ppm)
-3 -2 -1 0 1 2 3 4 5 6 7 8 9 10
f1 (ppm)

Lanthanum triple decker 10

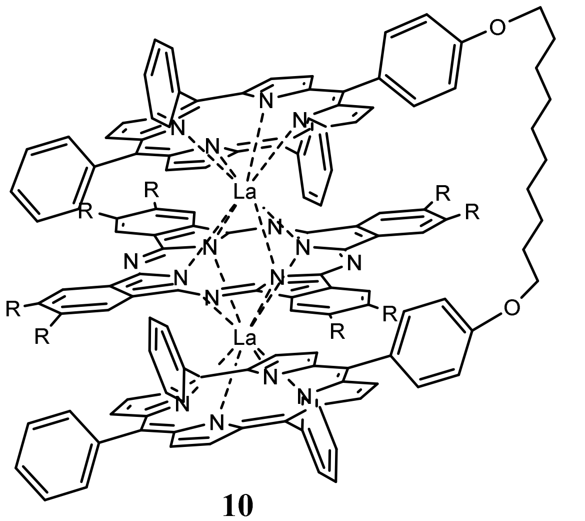

R = n-octyl

10

Porphyrin dyad **5** (9.9 mg, 7.1x10^{-3} mmol) was mixed with lanthanum(III) acetylacetonate hydrate (6.5 mg, 14.9x10^{-3} mmol) and dissolved in octanol (5 mL). The mixture was heated at reflux under Ar for 6 h and after complete metallation (checked by UV-vis), octa-*n*-octylphthalocyanine **8** (10 mg, 7.08x10^{-3} mmol) was added and reaction refluxed for 16h under Ar. The solvent was removed by distillation under reduced pressure to obtain a green solid that was purified by column chromatography using DCM:Pet ether (1:1 v/v) to obtain triple decker **10** (18.4 mg, 83 %). ^{1}H NMR (500 MHz, CD_2Cl_2) δ 10.04-9.96 (m, 8H) $H_{oiPh'}$, 9.12 (s, 2H) H_{ArpC}, 9.11 (s, 2H) H_{ArpC}, 9.10 (s, 2H) H_{ArpC}, 9.07 (s, 2H) H_{ArpC}, 8.45-8.40 (m, 6H) H_{ooPh}, 7.98 (d, *J* = 6.0 Hz, 2H) $H_{ooPh'}$, 7.81 (t, *J* = 8.0 Hz, 6H) H_{miPh}, 7.40 – 7.15 (m, 22H) H_{β}, H_{miPh} and H_{pPh}, 6.87 (d, *J* = 6.0 Hz, 2H) $H_{miPh'}$, 6.80 (d, *J* = 8.0 Hz, 2H) $H_{moPh'}$, 6.75 (t, *J* = 6.0 Hz, 6H) H_{moPh}, 4.53 (t, *J* = 7.0 Hz, 4H) -O-CH_2-, 3.43 – 3.26 (m, 16H) -CH_2-$_{Pc}$, 2.25 (t, *J* = 7.0 Hz, 4H) -CH_2-$_{por}$, 2.18 – 2.02 (m, 16H) -CH_2-$_{Pc}$, 1.97 – 1.77 (m, 27H) -CH_2-, 1.74 – 1.63 (m, 16H) -CH_2-$_{Pc}$, 1.38 – 1.19 (m, 38H) -CH_2-, 1.10 – 1.00 (m, 19H), 0.96 (t, *J* = 7.0 Hz, 6H), 0.87 (t, *J* = 7.0 Hz, 10H). MS (MALDI-tof): m/z = 3086.15 [M^+]. UV-vis, (DCM)/nm(log ε): 365(5.5), 421(5.7), 497(3.8), 610(3.6). IR (KBr, cm^{-1}): 2958, 2924, 2854, 1610, 1514, 1466, 1323, 1243, 1078, 984. (^{13}C NMR spectra could not be obtained due to slow decomposition in the NMR solvent over the long acquisition time).

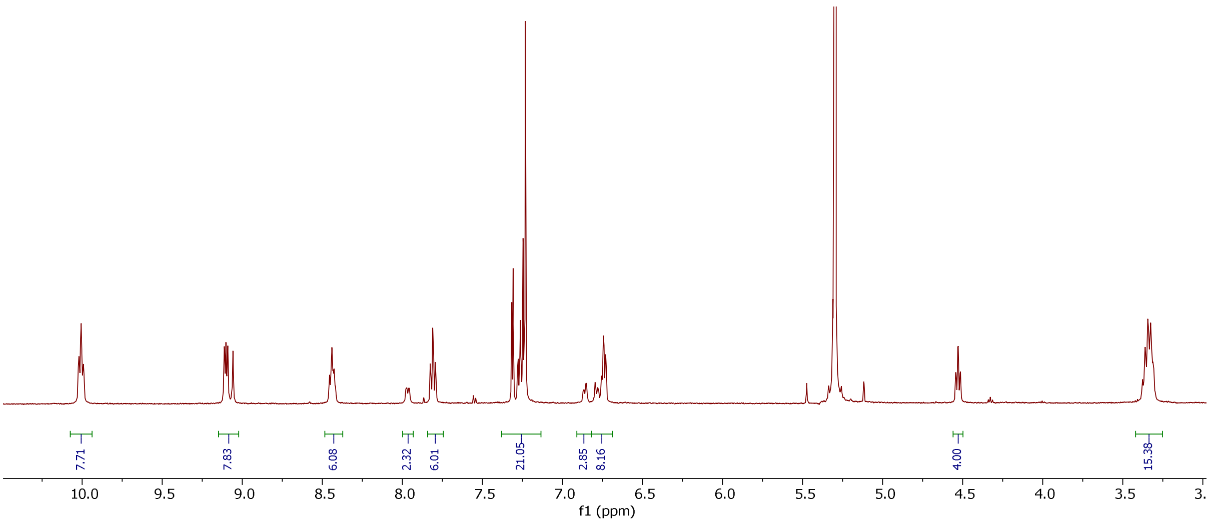

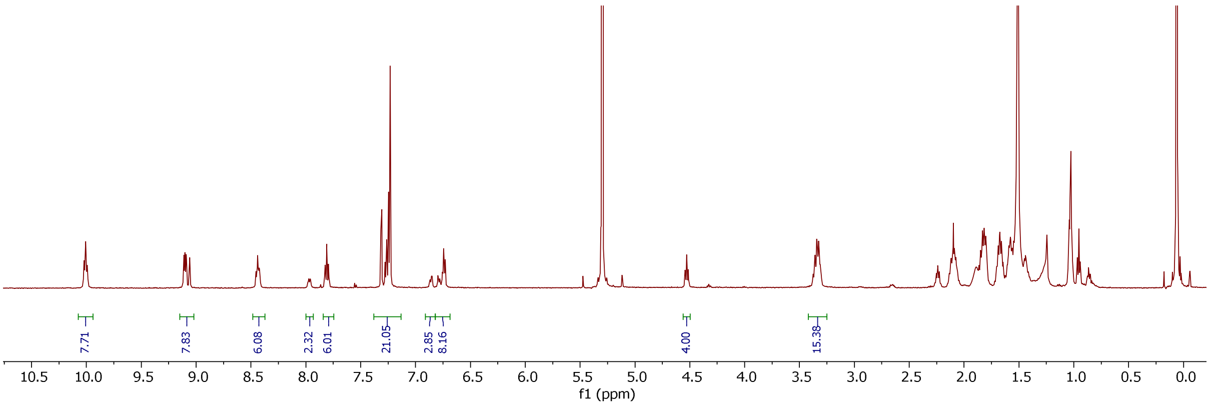

Lanthanum triple decker 11

5

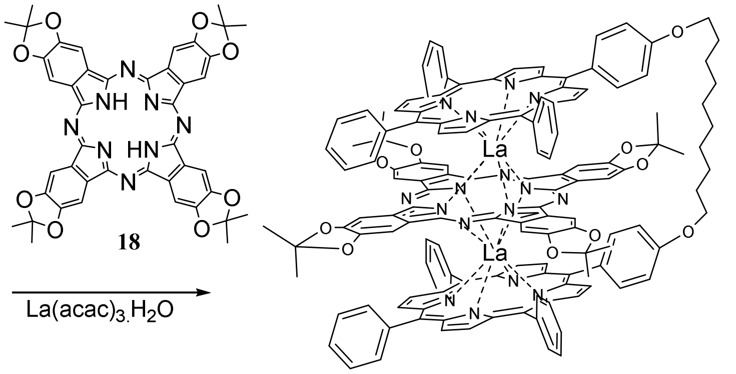

11

Porphyrin dyad **5** (30 mg, 0.021 mmol) was mixed with lanthanum(III) acetylacetonate hydrate (19.26 mg, 0.044 mmol) and dissolved in octanol (3 mL). The mixture heated at reflux under Ar for 6h, then phthalocyanine **18** (16 mg, 0.021 mmol) was added. After 18h the solvent was removed by distillation under reduced pressure and the crude washed with MeOH. The resulting brown solid was then separated by column chromatography through silica gel using EtOAc:Pet ether (1:4 v/v), collecting triple decker **11** as a dark brown solid (34.4 mg, 76 %). ^{1}H NMR (500 MHz, d-toluene) δ 10.41 (d, J = 8.5 Hz, 2H) $H_{oiPh'}$; 10.30 (d, J

= 7.0 Hz, 4H) H_{oiPh}; 10.25 (d, J = 7.0 Hz, 2H) $H_{oiPh'}$; 9.04 (s, 2H) H_{pC}; 9.02 (s, 2H) H_{pC}; 9.02 (s, 2H) H_{pC}; 8.99 (s, 2H) H_{pC}; 8.41 (t, J = 7.5 Hz, 6H) H_{ooPh}; 8.19 (d, J = 8.5 Hz, 2H) $H_{ooPh'}$; 7.80 (d, J = 4.5 Hz, 4H) H_{β}; 7.69 (d, J = 4.5 Hz, 4H) H_{β}; 7.67 – 7.64 (m, 8H) H_{β}; 7.60 (t, J = 7.5 Hz, 6H) H_{pPh}; 7.06 (d, J = 7.5 Hz, 6H) H_{miPh}; 6.92 (m, 2H) $H_{miPh'}$; 6.86 (m, 2H) $H_{moPh'}$; 6.77 (d, J = 6.5 Hz, 6H) H_{moPh}; 4.25 (t, J = 7.0 Hz, 4H) -O-CH_2-; -CH_2- (obscured by solvent); 1.64 (s, 12H) C-(CH_3)$_2$; 1.60 (s, 12H) C-(CH_3)$_2$. ^{1}H NMR (500 MHz, CD_2Cl_2) δ 9.90 (d, J = 8.5 Hz, 6H); 9.83 (d, J = 7.5 Hz, 2H) H_{oiPh}; 8.64-8.60 (4 x br s, 8H) H_{pC}; 8.45 (t, J = 7.5 Hz, 6H); 7.99 (d, J = 7.5 Hz, 2H); 7.84 (t, J = 7.0 Hz, 6H); 7.43 (d, J = 7.5 Hz, 4H); 7.37 – 7.34 (m, 8H); 7.60 (t, J = 7.5 Hz, 6H); 7.28 (t, J = 7.5 Hz, 6H); 6.86 (br d, 2H) $H_{miPh'}$; 6.86 (m, 2H); 6.72 (br t, 6H); 4.60 (t, J = 7.0 Hz, 4H) -O-CH_2-; 2.29-2.25 (br m, 4H), 1.89-1.85 br m, 8H), 1.78-1.74 (br m, 4H); 2.14 (s, 12H) C-(CH_3)$_2$; 2.10 (s, 12H) C-(CH_3)$_2$. ^{13}C NMR (126 MHz, CD_2Cl_2) δ 151.57, 148.06, 134.00, 128.95, 128.87, 128.76, 127.74, 126.49, 126.44, 120.61, 120.55, 30.26, 29.83, 29.13, 26.50, 26.43. MS (MALDI-tof): m/z = 2503.72 [M^+]. UV-vis, (DCM)/nm (log ε): 369(4.4), 419(4.8), 556(3.5), 602(3.5). IR (KBr, cm^{-1}): 2965, 2920, 2851, 1605, 1471, 1396, 1261, 1066, 982, 862.

Spectra in CD_2Cl_2

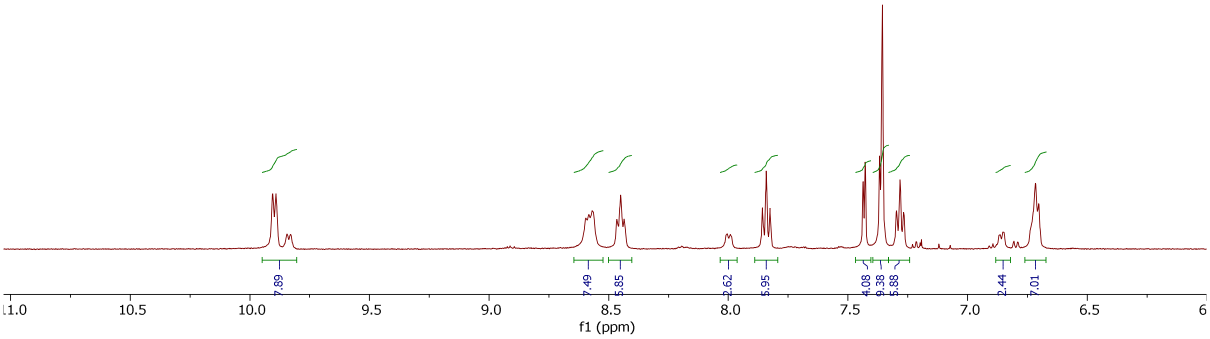

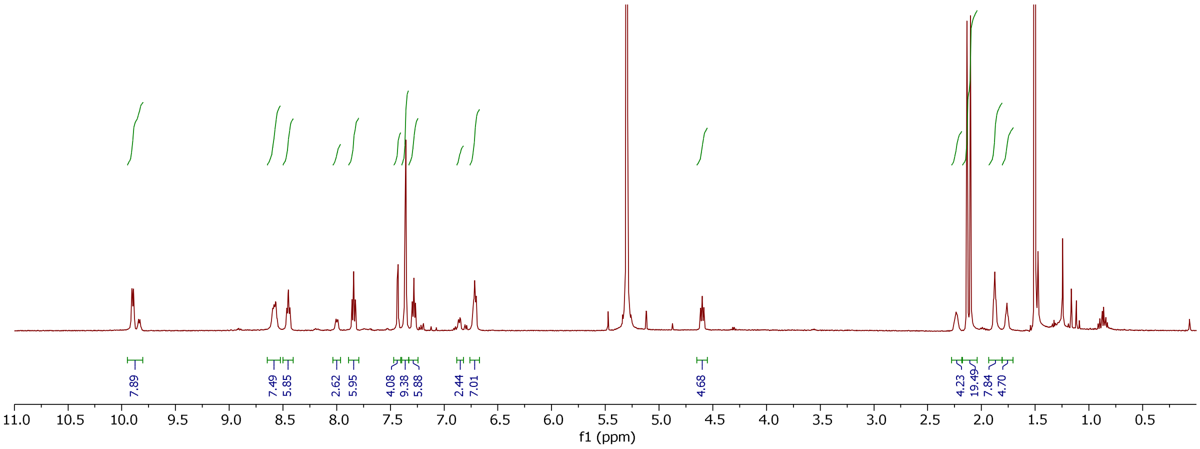

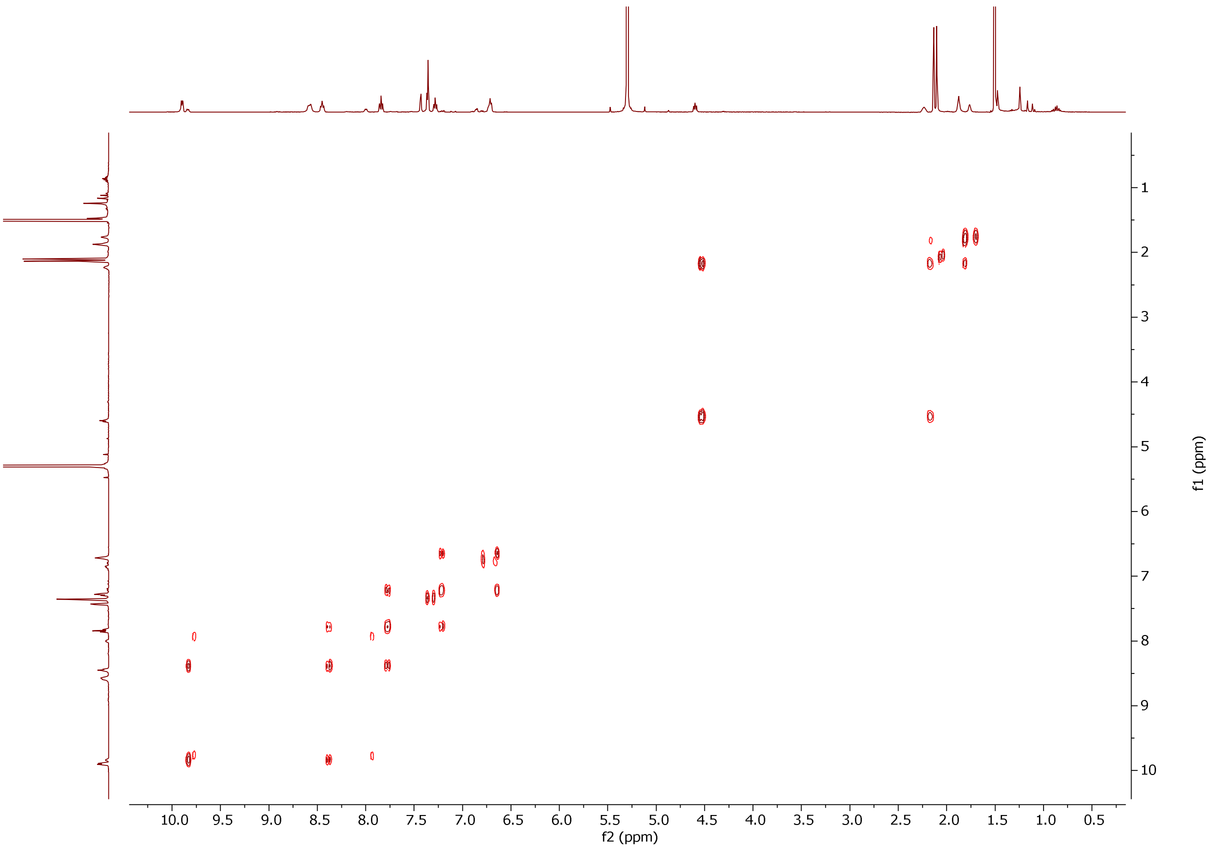

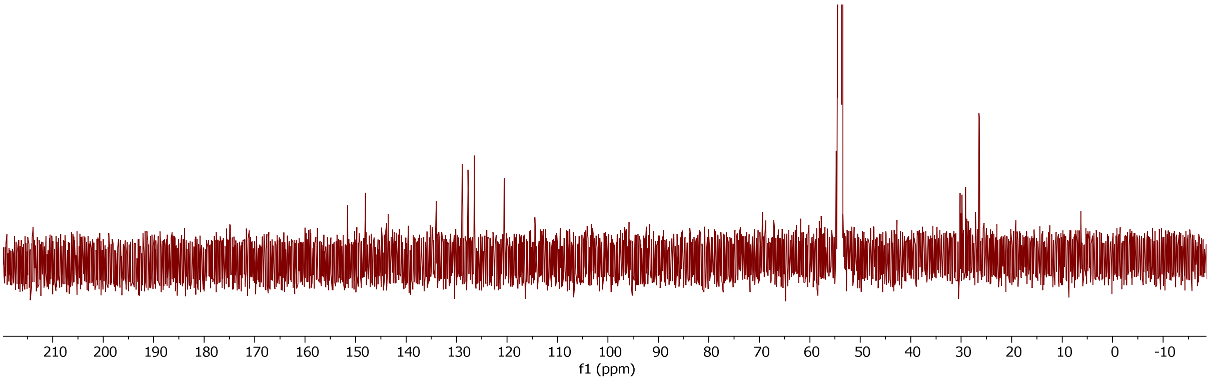

Spectra in d_6-Toluene

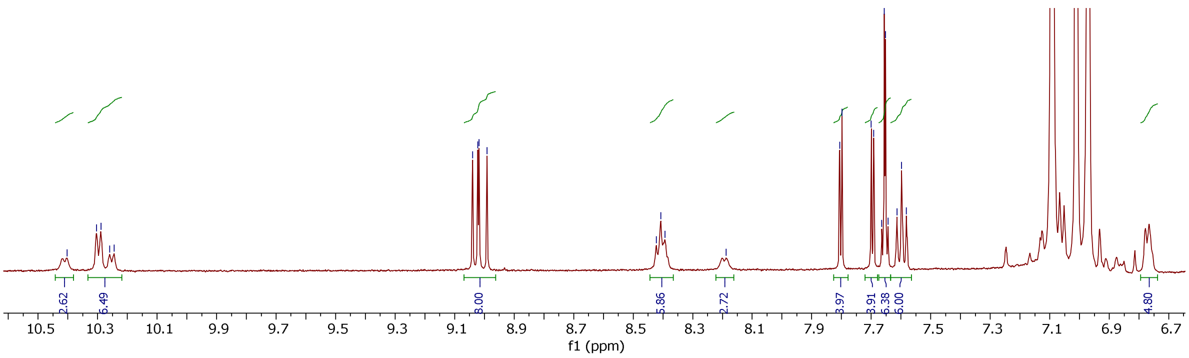

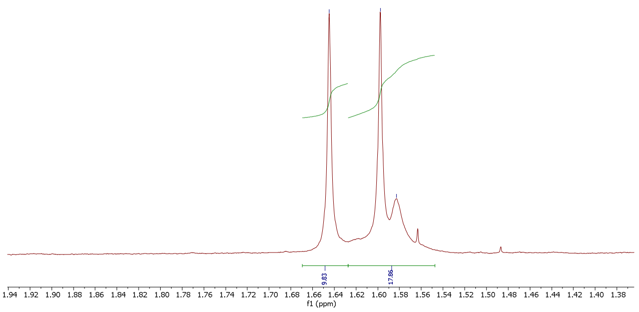

9.83
17.86
1.94 1.92 1.90 1.88 1.86 1.84 1.82 1.80 1.78 1.76 1.74 1.72 1.70 1.68 1.66 1.64 1.62 1.60 1.58 1.56 1.54 1.52 1.50 1.48 1.46 1.44 1.42 1.40 1.38
f1 (ppm)

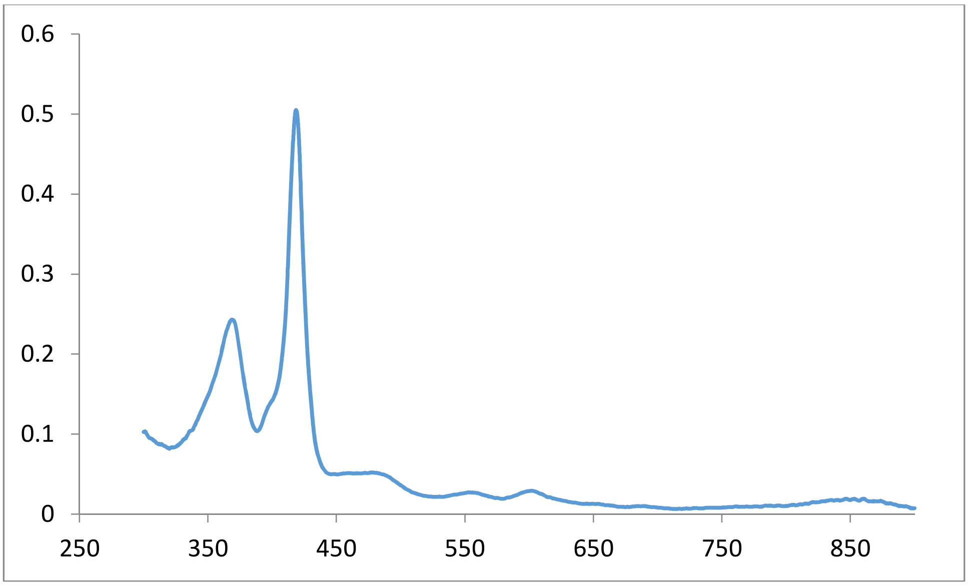

0.6
0.5
0.4
0.3
0.2
0.1
0
250 350 450 550 650 750 850

Crystallography

The structure crystallised in the triclinic P-1 space group with the tethered porphyrin unit and phthalocyanine disordered over two positions in equal proportions in the crystal. The solution is a superposition of both disorder components with a pseudo inversion centre at the centre of the middle phthalocyanine moiety of the assembly.

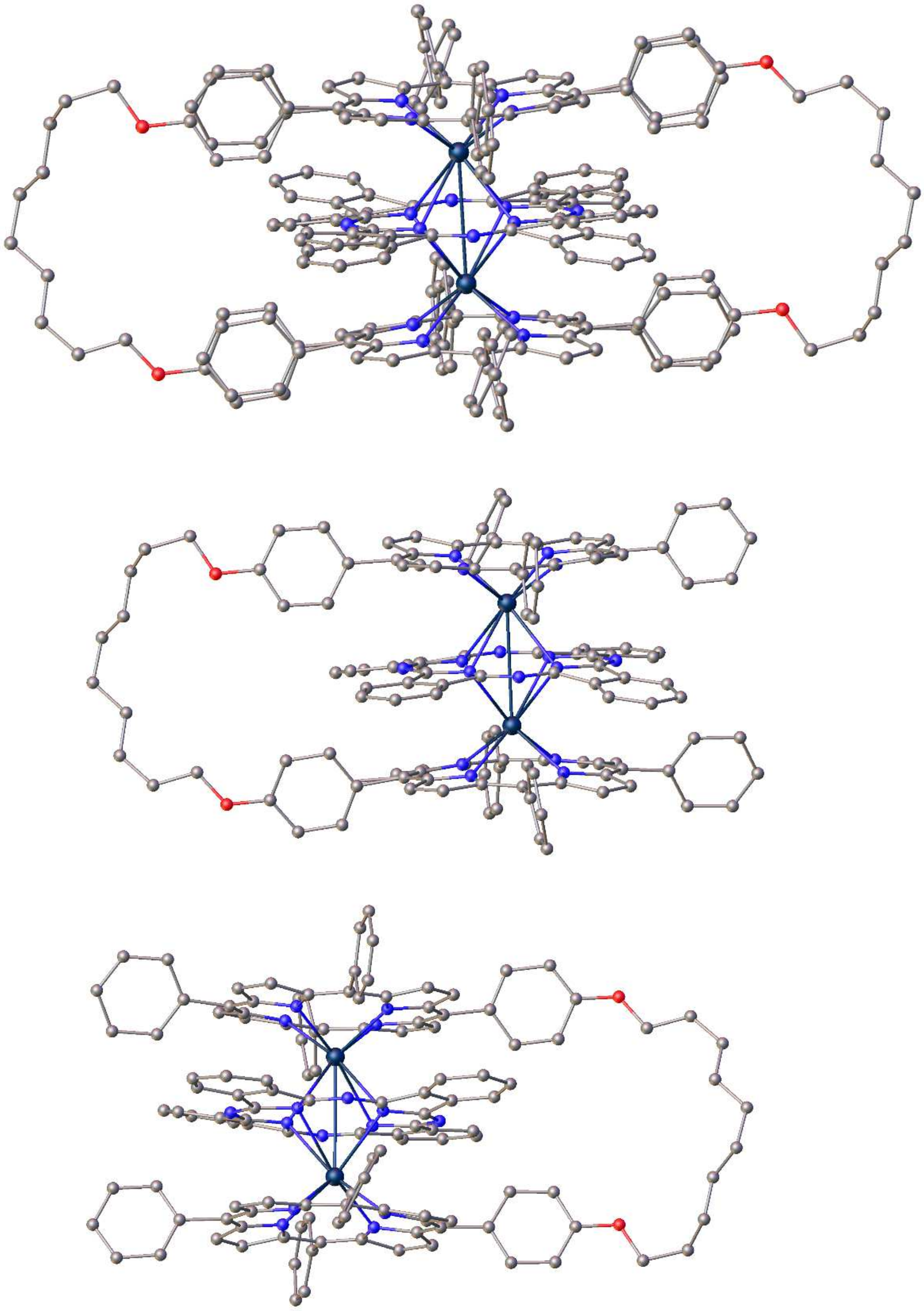

Crystallographic solution (top) is superposition of two conformations (middle and bottom) shown. Hydrogen atoms are omitted for clarity

Experimental. Single plate-shaped crystals of **7** were supplied. A suitable crystal 0.07×0.05×0.01 mm^3 was selected and mounted on a MITIGEN holder in oil on a Rigaku FRE+ equipped with VHF Varimax confocal mirrors and an AFC12 goniometer and HG Saturn 724+ detector. The crystal was kept at a steady T = 100(2) K during data collection. The structure was solved with the **ShelXT** (Sheldrick, 2015) structure solution program using the using dual methods solution method and by using **Olex2** (Dolomanov et al., 2009) as the graphical interface. The model was refined with version 2018/3 of **ShelXL** (Sheldrick, 2015) using full matrix least squares minimisation on $\boldsymbol{F}^2$ minimisation. All non-hydrogen atoms were refined anisotropically. Hydrogen atom positions were calculated geometrically and refined using the riding model. Solvent masking was used to eliminate the electron contribution equivalent to 3 EtOH and 1 MeOH molecules per unit cell. 1,2 and 1,3 equal distance restraints applied to equivalent atom pairs in each disorder component. Thermal restraints applied to all atoms. Geometrical and distance restraints applied to 'tethered' rings

Crystal Data. $C_{130}H_{90}La_2N_{16}O_2$, M_r = 2185.99, triclinic, P-1 (No. 2), a = 13.3123(6) Å, b = 14.4258(6) Å, c = 16.0910(8) Å, α = 83.466(4)°, β = 70.802(4)°, γ = 88.292(4)°, V = 2899.2(2) Å^3, T = 100(2) K, Z = 1, Z' = 0.5, μ(Mo K_α) = 0.784 mm^{-1}, 29649 reflections measured, 10148 unique (R_{int} = 0.0749) which were used in all calculations. The final wR_2 was 0.1967 (all data) and R_1 was 0.0718 (I > 2(I)).
